# Landscape of potentially targetable receptor tyrosine kinase fusions in diverse cancers by DNA-based profiling

**DOI:** 10.1038/s41698-022-00325-0

**Published:** 2022-11-11

**Authors:** Tiantian Wang, Li Wei, Qiang Lu, Yanmei Shao, Shuqing You, Jiani C. Yin, Sha Wang, Yang Shao, Zhanhong Chen, Zhe Wang

**Affiliations:** 1 Department of Medical Oncology and Guangdong Key Laboratory of Liver Disease, The Third Affiliated Hospital of Sun Yat-sen University, Guangzhou, 510000 China; 2 Department of Thoracic Surgery, Tangdu Hospital, The Air Force Military Medical University, Xi’an, 710038 China; 3 Department of Respiratory and Critical Care Medicine, The Affiliated Hospital of Qingdao University, Qingdao, 266000 China; 4 Department of Pathology, Taizhou First People’s Hospital, Taizhou, Zhejiang 318020 China; 5 Geneseeq Research Institute, Nanjing Geneseeq Technology Inc., Nanjing, Jiangsu 210031 China; 6 School of Public Health, Nanjing Medical University, Nanjing, Jiangsu 210029 China; 7 Department of Thoracic Surgery, National Cancer Center/National Clinical Research Center for Cancer/Cancer Hospital & Shenzhen Hospital, Chinese Academy of Medical Sciences and Peking Union Medical College, Shenzhen, 518116 China

**Keywords:** Cancer genomics, Next-generation sequencing

## Abstract

Recurrent fusions of receptor tyrosine kinases (RTKs) are often driving events in tumorigenesis that carry important diagnostic value and are potentially targetable by the increasing number of tyrosine kinase inhibitors (TKIs). Here, we characterized the spectrum of 1324 RTK fusions with intact kinase domains in solid tumors by DNA-based high-throughput sequencing. Overall, the prevalence of RTK fusions were 4.7%, with variable frequencies and diverse genomic structures and fusion partners across cancer types. Cancer types, such as thyroid cancers, urological cancers and neuroendocrine tumors are selective in the RTK fusions they carry, while others exhibit highly complex spectra of fusion events. Notably, most RTKs were promiscuous in terms of the partner genes they recombine with. A large proportion of RTK fusions had one of the breakpoints localized to intergenic regions. Comprehensive genomic profiling revealed differences in co-mutational patterns pre- and post-TKI treatments across various RTK fusions. At baseline, multiple cases were detected with co-occurring RTK fusions or concomitant oncogenic mutations in driver genes, such as *KRAS* and *EGFR*. Following TKI resistance, we observed differences in potential on- and off-target resistance mutations among fusion variants. For example, the *EML4*-*ALK* v3 variant displayed more complex on-target resistance mechanisms, which might explain the reduced survival outcome compared with the v1 variant. Finally, we identified two lung cancer patients with *MET*+ and *NTRK1*+ tumors, respectively, who responded well to crizotinib treatment. Taken together, our findings demonstrate the diagnostic and prognostic values of screening for RTK fusions using DNA-based sequencing in solid tumors.

## Introduction

Many fusion genes are drivers of tumorigenesis, and have important diagnostic and prognostic values in informing clinical action^1^. In particular, fusions of receptor tyrosine kinases (RTKs) represent an important class of oncogenic events that are selected for during cancer initiation and progression. Although found at a lower rate in solid tumors compared with hematologic malignancies, large-scale genomic studies have identified important RTK fusions across a wide range of cancer types^2–5^. Given the potential druggability of RTKs, extensive characterization of the landscape of RTK fusions would likely facilitate new drug development and expand the therapeutic options for cancer patients.

Traditional methods, such as fluorescence in situ hybridization (FISH) and quantitative real-time polymerase chain reaction (RT-PCR) or PCR, are highly sensitive in detecting fusion genes. However, such low-throughput methods are time- and cost-ineffective and also suffer from its limitations in detecting rare fusion events. Recent advances in massively parallel sequencing and bioinformatics methods have revealed the complexity of genetic fusions in cancer. A number of genomic approaches have been commonly applied for the detection of fusion events, including whole genome sequencing (WGS), RNA sequencing (RNA-seq), and targeted DNA sequencing. However, in the clinical setting, targeted DNA sequencing has its unique advantages in increased sensitivity and also overcoming the challenges of fusion detection using formalin-fixed paraffin-embedded (FFPE)-derived tumors.

In this study, we sought to characterize the prevalence and the spectrum of RTK fusions in patients with diverse solid tumors who underwent hybridization capture-based DNA-targeted sequencing. We also examined co-mutations and potential resistance mechanisms at baseline and following TKI treatment, respectively.

## Results

### Prevalence of RTK fusions across diverse cancers

We examined the frequencies of RTK fusions in a large cohort of Chinese patients across a diverse range of solid tumor types (Supplementary Fig. 1) whose tumor and/or ctDNA samples underwent targeted profiling. Only those fusion events that retained the intact kinase domain were included in the analysis.

**Fig. 1:**
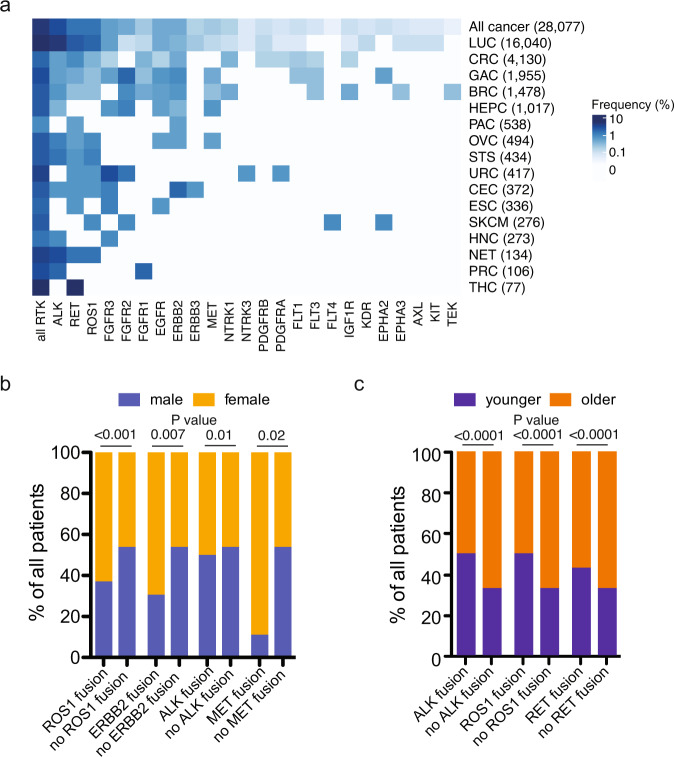
Landscape of RTK fusions across diverse cancers. **a** Heatmap showing the prevalence of rearrangements of specific RTKs in different cancers, LUC lung cancer, CRC colorectal cancer, GAC gastric cancer, BRC breast cancer, HEPC hepatobiliary cancer, PAC pancreatic cancer, OVC ovarian cancer, STS soft tissue sarcoma, CEC cervical cancer, ESC esophageal cancer, URC urinary cancer, HNC head and neck cancer, SKCM skin cutaneous melanoma, NET neuroendocrine tumor, PRC prostate cancer, THC thyroid cancer. **b** *ROS1*, *ERBB2*, *ALK*, and *MET* fusions showed increased associations with the female sex. **c** *ALK*, *ROS1*, and *RET* fusions showed increased associations with younger age.

The overall prevalence of RTK fusions detected in this cohort was 4.7% (*n* = 1324), with varying frequencies across different cancer types (Fig. 1a and Table S1). RTK fusions were detected at high frequencies in thyroid cancers (7.8%), lung cancers (7.1%), neuroendocrine tumors (3.7%), urological cancers (3.6%), and gastric cancers (2.2%). In line with previous reports, our analysis recapitulated the key targetable oncogenic fusion events in lung cancers, with a 4.2% frequency of *ALK* fusions, 1.3% of *RET* fusions, and 1.2% of *ROS1* fusions (Fig. 1a and Supplementary Table 1). Multiple additional but relatively rare oncogenic fusions have been described in lung cancers, including fusions of the FGFR, NTRK, MET, and ErbB family genes^6–8^. In our lung cancer cohort, we also observed a 0.2% of *FGFR* family (mostly *FGFR3*) fusions and 0.02% of *NTRK1*/*3* gene fusions. Rearrangements of *MET* and the ErbB family RTKs (*EGFR*, *ERBB2*, *ERBB3*, and *ERBB4*) in lung cancer are less well documented and mostly as case reports^6,9^. A total of three (0.02%) lung cancer patients harbored *MET* fusions (Fig. 1a and Supplementary Table 1), against which crizotinib has reportedly demonstrated clinical activity^10,11^. On the other hand, the prevalence of ErbB family gene fusions in our lung cancer cohort was non-negligible reaching 0.23%, with the majority of these patients carrying *EGFR* (0.11%) and *ERBB2* (0.07%) fusions.

Besides lung cancer, several other cancer types also displayed a broad array of RTK fusions, particularly gastric cancers, colorectal cancers and breast cancers (Fig. 1a and Supplementary Table 1). The top oncogenic RTK fusions in gastric cancer were of the FGFR family genes (total, 1.4%; *FGFR2*, 1.2%) and ErbB family genes (total, 0.67%; *ERBB2*, 0.41%, *EGFR*, 0.26%), with a smaller percentage of patients also harboring *EPHA2* (0.15%), *ALK* (0.15%), *MET* (0.10%), *RET* (0.10%), and *ROS1* (0.07%) fusions. The overall prevalence of RTK fusions in colorectal cancers was 1.0%, with the top frequent fusion genes being *RET* (0.34%), *ALK* (0.19%), and *NTRK1* (0.10%). In breast cancers, the most prevalent fusion genes were FGFR family genes (total, 0.48%; *FGFR2*, 0.34%; *FGFR1*, 0.14%), ErbB family genes (total, 0.47%; *EGFR*, 0.20%; *ERBB2*, 0.27%), *ALK* (0.27%) and *ROS1* (0.07%).

By contrast, a restricted spectrum of RTK fusions was observed in thyroid cancers, urological cancers, and neuroendocrine tumors. Of the 77 patients with thyroid cancers, six (7.8%) RTK fusion events were identified, which were exclusively *RET* fusions. Urological cancers also had a high proportion (15 out of 417; 3.6%) of RTK fusions, with the majority (12; 80%) of patients carrying *FGFR2*/*3* fusions. Similarly, *ALK* fusions (2.2%) accounted for three of the five fusion-positive cases with neuroendocrine tumors.

RTK fusions were detected in both the tumor specimens (*n* = 963) and circulating cell-free DNA (cfDNA) samples from a variety of body fluids, including plasma (*n* = 268), pleural effusion (*n* = 79), cerebrospinal fluid (*n* = 6), and additional liquid biopsy samples from ascites and pericardial effusions (*n* = 8). No apparent differences were observed between the overall frequencies detected in the tumor and cfDNA samples (Supplementary Fig. 2A and Supplementary Tables 2–3). In addition, comparing the RTK frequencies in lung cancer between tumor specimens and cfDNA samples, only a slight enrichment of *RET* fusions in the cfDNA samples were detected (false discovery rate (FDR) adjusted *q* = 0.04; Supplementary Fig. 2B). No other significant differences were identified.

Significant associations of sex and age with the occurrence of specific RTK fusions were also observed. Overall, *ROS1* (63% vs. 46%, Fisher’s exact test *P* < 0.0001, FDR-adjusted *q* = 0.001), *ERBB2* (69% vs. 46% Fisher’s exact test *P* = 0.007, FDR-adjusted *q* = 0.04), *ALK* (50% vs. 46%, Fisher’s exact test *P* = 0.01, FDR-adjusted *q* = 0.05), and *MET* (89% vs. 46%; Fisher’s exact test *P* = 0.02, FDR-adjusted *q* = 0.06) fusions were more frequently detected in females (Fig. 1b). Taking into account potential cancer type differences, we found that *ROS1* (63% vs. 44%; Fisher’s exact test *P* < 0.0001) and *ALK* fusions (50% vs. 44%, Fisher’s exact test *P* = 0.006) remain more prevalent in female lung cancer patients (Supplementary Fig. 3A). No differences were found comparing the sex distributions among different variants of *ALK* (Supplementary Fig. 3B), *RET* (Supplementary Fig. 3C) or *ROS1* (Supplementary Fig. 3D). In addition, fusions in *ALK* (overall, Fisher’s exact test *P* < 0.0001; lung cancer, *P* < 0.0001), *ROS1* (overall, Fisher’s exact test *P* < 0.0001; lung cancer, *P* < 0.0001) and *RET* (overall, Fisher’s exact test *P* = 0.006; lung cancer, *P* < 0.0001) were associated with an earlier disease onset (Fig. 1c and Supplementary Fig. 4A). Further analysis stratified age differences in *ALK* fusion variants and showed that the *EML4*-*ALK* v1 variant was associated with an earlier disease onset (Fisher’s exact test *P* = 0.008), while the v3 variant was more likely to occur at an older age (Fisher’s exact test *P* = 0.02, Supplementary Fig. 4B). No age differences among *ROS1* or *RET* fusion variants were found (Supplementary Fig. 4C, D).

### Genomic structures and partner genes of RTK fusions

**Fig. 2:**
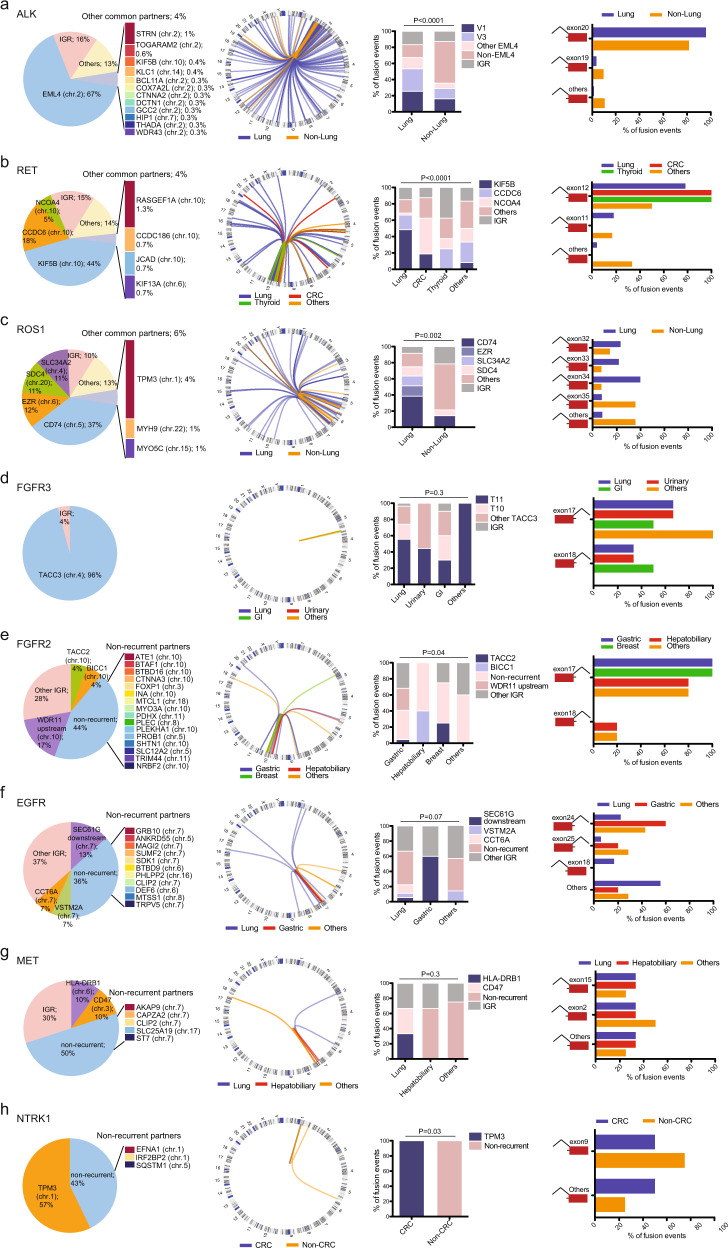
Genomic structures of the common RTK fusion genes. **a**–**h** From left to right: Distributions of common fusion partners; Circos plot showing the genomic rearrangement events in different cancer types; Comparisons of fusion partner frequencies in different cancer types, *P* values using chi-square tests are as indicated; Comparisons of RTK breakpoints in different cancer types in **a** *ALK*, **b** *RET*, **c** *ROS1*, **d** *FGFR3*, **e** *FGFR2*, **f** *EGFR*, **g** *MET*, and **h** *NTRK1* positive samples. IGR intergenic regions.

The genomic structures and partner genes of the most commonly altered RTK fusion genes (*ALK*, *RET*, *ROS1, FGFR3/2*, *EGFR*, *MET*, and *NTRK1*) were illustrated in Fig. 2. While *EML4* accounted for 66.5% (610/917) of the *ALK* fusion events in this cohort, a remarkably diverse array of *ALK* fusion partners were detected (Fig. 2a). A total of 227 distinct fusion partners of *ALK* were detected. Among these, some recurrent *ALK* partners included *STRN* (1.0%), *TOGARAM2* (0.5%), *KIF5B* (0.4%), and *KLC1* (0.4%), and 46 fusion partners occurred only once. In addition, there were a considerable number (15.4%, 141/917) of intergenic rearrangements (i.e., having one breakpoint localized to intergenic regions (IGR)). The *EML4*-*ALK* fusion gene is generated by an inversion on chromosome 2^12^. For the majority (65.5%, 201/307) of non-*EML4* fusions, including 74% of intergenic rearrangements, they were also clustered on chromosome 2. Notably, recombination of *ALK* could occur with a vast range of chromosomal regions with fusion partners scattered across the genome (Fig. 2a). *ALK* fusions in non-lung cancer patients were more commonly fused with non-*EML4* partners (Bonferroni’s post-test, *P* < 0.0001; Fig. 2a). Of the 31 *ALK* fusion events detected in non-lung cancers, including gastrointestinal and gynecological cancers, 11 patients (35.5%) were detected with *EML4*-*ALK* fusions. *STRN* represented the second most common *ALK* fusion partner, and were detected in two cases of colorectal cancer and one case of hepatobiliary cancer. All *ALK* fusions took place at the 5’ end of the protein and breakpoints in intron 19 were highly recurrent, accounting for 95% overall, 96% in lung cancers and 81% in non-lung cancers, producing fusion products fused to exon 20 of the *ALK* gene (Fig. 2a). No clear consensus sequences were found around the breakpoints of the *ALK* gene across different cancer types (Supplementary Fig. 5A, B) or fusion variants (Supplementary Fig. 5C–F). Consistent with v1 and v3 being the most common *ALK* fusion variants, breakpoints in *EML4* mostly clustered around exons 6 and 13 (Supplementary Fig. 5G). Other recurrent breakpoints included exon 3 of *STRN* and 5’UTR of *TOGARAM2* (Supplementary Fig. 5H). Interestingly, the breakpoints of *ALK* fusion partners, *EML4* or non-*EML4*, were surrounded by AT-rich regions (Supplementary Fig. 5I, J).

Fifty distinct *RET* 5’ fusion partners were identified, with *KIF5B* being the most common fusion partner, accounting for 44% (131/298) of *RET* fusions, which was followed by *CCDC6* (52/298, 18%) and *NCOA4* (16/298, 5%; Fig. 2b). Other recurrent fusion partners included *RASGEF1A* (1.3%), *CCDC186* (0.7%), *JCAD* (0.7%), and *KIF13A* (0.7%; Fig. 2b). All recurrent fusion partners of *RET*, except for *JCAD*, has been previously reported. Of the two *JCAD*-*RET* cases, one had lung adenocarcinoma and the other were diagnosed with soft tissue sarcoma of the cervix, and both had the 5’ UTR of *JCAD* fused to the *RET* gene. *RET* fusions were mostly caused by rearrangements with nearby genes or intergenic regions on chromosome 10 (Fig. 2b). In addition to lung cancers, *RET* fusions were also frequently detected in colorectal and thyroid cancers. The frequencies of the recurrent fusion partners differed across the cancer types (Fig. 2b). For example, *KIF5B*-*RET* was highly frequent but almost exclusively found in lung cancers (127/262, 48.5%; Bonferroni’s post-test, *P* < 0.001). It was found in only 18.8% (3/16) of colorectal cases and one case of neuroendocrine tumors, but none of the eight thyroid cases. On the other hand, while the frequency of *NCOA4*-*RET* was low (2.3%, 6/262) in lung cancer, it was the most frequent *RET* fusion (44%, 7/16) in colorectal cancer (Bonferroni’s post-test, *P* < 0.0001). In addition, *CCDC6*-*RET* was only detected in non-colorectal cancers. The most frequent breakpoints across all cancers took place in *RET* intron 11, resulting in a fusion product involving *RET* exon 12 (overall rate, 78.9%, 235/298). Across all *RET* fusions, regardless of cancer types (Supplementary Fig. 6A, B) or fusion variants (Supplementary Fig. 6C–E), GC-rich sequences were found around the breakpoints of *RET*. For the common *RET* fusion partners, breakpoints in *KIF5B*, *CCDC6* and *NCOA4* most commonly occurred in exons 15, 1, and 7, respectively (Supplementary Fig. 6F–H), and were enriched with AT-rich sequences (Supplementary Fig. 6I–K).

Unlike *ALK* and *RET*, whose fusion partners were largely nearby genes residing on the same chromosome, *ROS1* fusions were more scattered across the entire genome (Fig. 2c). Only 20.9% (48/230) of *ROS1* rearrangements were generated by translocations in chromosome 6. Overall, thirty-nine distinct *ROS1* fusion partners were detected. In lung cancers, fusions with *CD74* (83/216, 38.4%), *EZR* (28/216, 13.0%), *SDC4* (26/216, 12.0%), and *SLC34A2* (26/216, 12.0%) were highly frequent (Fig. 2c). Of these, the generation of *CD74*-*RET*, *SDC4*-*RET* and *SLC34A2*-*RET* fusions involved translocation with chromosome 5, 20, and 4, respectively. *TPM3*-*ROS1*, which was another relatively common form of *ROS1* fusions in lung cancers (8/216, 3.7%), was generated by translocation with chromosome 1. Other recurrent *ROS1* fusions included *MYH9*-*ROS1* (2/216, 0.9%) and *MYO5C*-*ROS1* (2/216, 0.9%), involving translocation with chromosome 22 and 15, respectively. In the non-lung cancer cases (*n* = 14), *ROS1* fusions mainly involved chromosome 5 and 6 (Fig. 2c). Non-lung cancers were more likely to harbor rare *ROS1* fusions (Bonferroni’s post-test, *P* = 0.002). *CD74*-*ROS1* was detected in one case each of cervical and urinary cancers, and *SDC4*-*ROS1* were detected in one case of soft tissue sarcoma. Similar to the wide genomic distributions of its fusion partners, breakpoints of *ROS1* also spanned across multiple introns, mostly from intron 31 to intron 34 (Fig. 2c). Unlike *ALK* or *RET*, the sequences around the breakpoints of *ROS1* were highly AT-rich (Supplementary Fig. 7A–F). For common *ROS1* partner genes, breakpoints in *CD74*, *EZR*, *SDC4* and *SLC34A2* were predominantly located in exons 6, 10, 2, 13, respectively (Supplementary Fig. 8A, B). *TPM3* breakpoints were mostly located in exons 7 and 8 (Supplementary Fig. 8B). Sequences around the breakpoints of *CD74* were GC-rich (Supplementary Fig. 8C), whereas sequences around all other *ROS1* partners tended to be AT-rich (Supplementary Fig. 8D–F).

FGFR and ErbB family fusions were also rather common in a multitude of cancers. Of the FGFR family, *FGFR2* and *FGFR3* fusions accounted for 39% and 51% of the total *FGFR* fusion events, respectively. *FGFR3* fusions were common in lung and urinary cancers, but were also found in a wide variety of cancer types, particularly cancers of the gastrointestinal tract (Fig. 2d). Remarkably, regardless of cancer types, *FGFR3* fusions were almost exclusively in the form of *FGFR3*-*TACC3* (94%, 47/50), generated by translocations in chromosome 4. Breakpoints in *FGFR3* were either in intron 17 or exon 18. The most frequent (19/47, 40.4%) breakpoints involved intron 17 of *FGFR3* and intron 11 of *TACC3*, resulting in the F17:T11 fusion variant. Regions around the breakpoints of *FGFR3* and *TACC3* were enriched with GC-rich sequences (Supplementary Fig. 9A–E). In contrast to *FGFR3*, fusion partners of *FGFR2* were highly diverse although mostly located on chromosome 10 (Fig. 2e). The two recurrent *FGFR2* fusions were *FGFR2*-*BICC1* in two cases of hepatobiliary cancers (Bonferroni’s post-test, *P* = 0.006) and *FGFR2*-*TACC2* in one case each of breast and gastric cancers. Interestingly, one recurrent intergenic *FGFR2* fusion was identified solely in patients with gastric cancer (6/22, 27.3%), being fused to intergenic regions upstream of *WDR11*. Similar to *FGFR3*, the majority of *FGFR2* breakpoints were located in intron 17. No specific sequence patterns were found around the breakpoints of *FGFR2* and its partner genes (Supplementary Fig. 9F–H).

Of the ErbB family, *EGFR* and *ERBB2* were the most frequently rearranged genes. The majority of ErbB fusions were non-recurrent and occurred downstream of the kinase domain. Recurrent *EGFR* fusions included *CCT6A*-*EGFR* fusions in two cases of lung cancer (2/13, 15.4%) and intergenic *EGFR*-*SEC61G* fusions, occurring at intergenic regions downstream of *SEC61G*, in three cases of gastric cancers and one case of lung cancer (Fig. 2f). *EGFR*-*VSTM2A* fusion was detected in one case of colorectal cancer and another case occurring upstream of the *VSTM2A* gene in lung cancer. Fusion partners were mostly localized to chromosome 7 (12/30, 40%), whereas breakpoints in *EGFR* were rather widely distributed as shown in Fig. 2f. The breakpoints for 3’ end *EGFR* fusions were predominantly in intron 24 (10/30, 33.3%) and for 5’end *EGFR* fusions, intron 17 (3/30, 10%; Fig. 2f). No clear consensus was found for the sequences around the breakpoints of *EGFR*, although those of its partner genes tended to be AT-rich (Supplementary Fig. 10A–C). Recurrent *ERBB2* fusions included *ERBB2*-*PGAP3* in a total of three cases of gastric, colorectal, and cervical cancers and *GRB7*-*ERBB2* in one case each of lung and cervical cancers. The majority of *ERBB2* rearrangements occurred in chromosome 17 (31/36, 86.1%). Similar to *EGFR*, fusions in *ERBB2* occurred at many breakpoints across the gene, with exon 27 being the most frequent spot (7/36, 19.4%). Regions around the *ERBB2* breakpoints were enriched with GC-rich sequences and no clear sequence consensus was observed near the breakpoints of its partner genes (Supplementary Fig. 10D, E).

*MET* and *NTRK* fusions occurred at relatively low frequencies, but were detected in a number of cancer types surveyed. *MET* partners were all fused to the 5’ end of the protein and mostly localized to chromosome 7 (Fig. 2g). No recurrent *MET* fusions were identified, some of the fusion partners included *HLA*-*DRB1*-*MET* (lung cancer), *CD47* (lung cancer), *CAPZA2* (gastric cancer), *AKAP9* (hepatobiliary cancer), *CLIP2* (hepatobiliary cancer), and *SLC25A19* (ovarian cancer). Of these, the *HLA*-*DRB1*-*MET* fusion and *CLIP2*-*MET* fusion were previously reported in cases of lung cancer^11^ and glioneuronal cancer^13^, respectively. For *NTRK* fusions, all of the four *NTRK1* + colorectal cases harbored a recurrent *TPM3*-*NTRK1* fusion (overall, 4/7, 57.1%; Bonferroni’s post-test, *P* = 0.04). Three non-recurrent *NTRK1* fusions were each detected in two cases of lung cancer (*SQSTM1*-*NTRK1* and *IRF2BP2*-*NTRK1*) and one case of breast cancer (*EFNA1*-*NTRK1*). Of these non-recurrent fusions, *SQSTM1*-*NTRK1* and *IRF2BP2*-*NTRK1* have been previously reported^14^. *NTRK3* fusions were detected in only two cases in the entire cohort, one *ABHD17C*-*NTRK3* fusion in a case with urinary cancer and one *TTC23*-*NTRK3* fusion in a lung cancer case. Although no clear consensus sequences were found in regions near the breakpoints of *MET* and *NTRK*, the *NTRK* gene showed marked enrichment of the C base at the location of the breakpoint (Supplementary Fig. 10F–I).

### Analysis of co-mutations in RTK fusion-carriers prior to TKI treatment

**Fig. 3:**
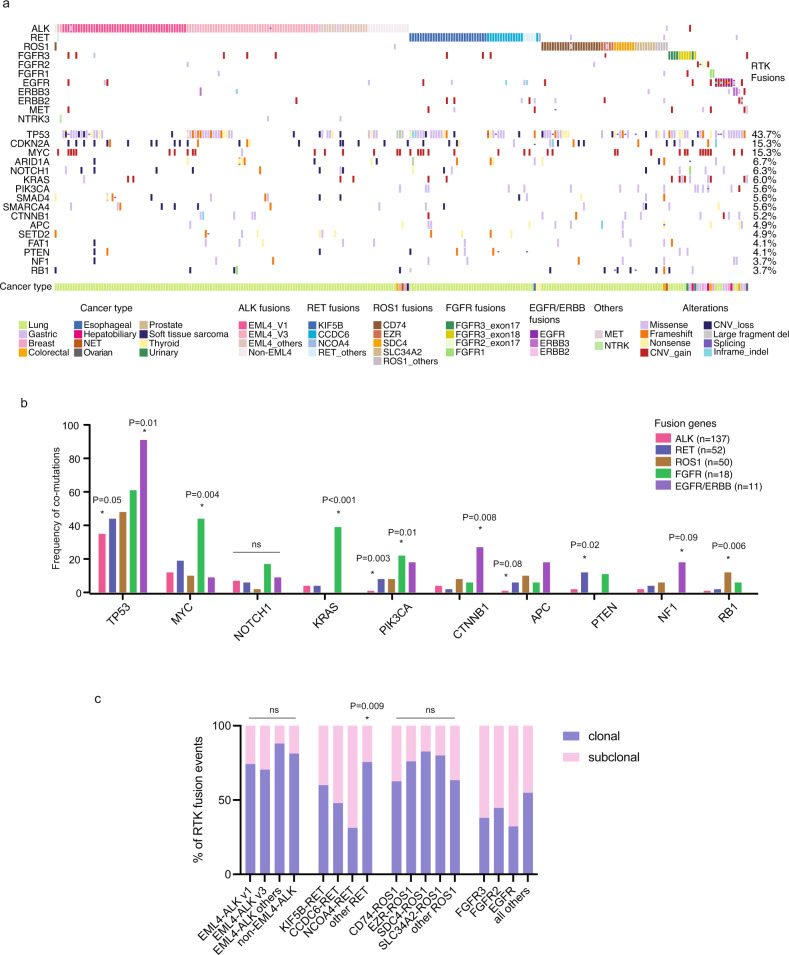
Mutational landscape of concomitant mutations prior to TKI treatment. **a** Oncoplot showing the most frequently co-mutated genes across different RTK fusion-positive samples. **b** Different RTK fusions are associated with varying spectra of concomitant mutations, *P* values using Bonferroni’s post-test are as indicated. **c** Estimated clonality comparing different fusion genes or fusion variants.

For patients with sufficient clinical records, including treatment regimens and timelines, we next investigated the mutations co-occurring with the respective RTK fusions prior to and following targeted TKI treatments. The top genes frequently co-mutated with RTK fusions prior to TKI treatment were illustrated in Fig. 3a and Supplementary Fig. 11. At the level of each RTK genes, we found that fusions in *EGFR*, *ERBB2* and *MET* were highly likely to be accompanied by an amplification of the respective RTK gene. Specifically, copy number gain occurred in all cases of *EGFR* and *ERBB2* fusion-positive cases, except for one case of *EGFR* fusion+ hepatobiliary cancer. The most frequently altered gene was *TP53*, with varying frequencies across different RTK fusion genes (Fig. 3a, b). *ALK* fusions were associated with a relatively lower frequency of *TP53* co-mutations (35%, Bonferroni’s post-test, *P* = 0.05), whereas over 90% of the ErbB family fusions carried an additional *TP53* mutation (Bonferroni’s post-test, *P* = 0.01).

In addition, different RTK fusions showed substantial differences in the pattern of their concomitant mutations (Fig. 3b). The overall co-mutation rates in *ALK* + cases were low, with significantly lower frequencies of *PIK3CA* (1%, Bonferroni’s post-test, *P* = 0.003) and *APC* (1%, Bonferroni’s post-test, *P* = 0.08) alterations compared with other RTK fusion+ cases. *RET* fusions were associated with a higher rate of *PTEN* alterations (12%, Bonferroni’s post-test, *P* = 0.02), whereas *ROS1* fusions were associated with a higher rate of *RB1* (12%, Bonferroni’s post-test, *P* = 0.006) in comparison with other RTK fusion+ cases. *FGFR* fusions were characterized by high frequencies of *PIK3CA* (22%, Bonferroni’s post-test, *P* = 0.01) and *KRAS* (39%, Bonferroni’s post-test, *P* < 0.001) alterations. Finally, ErbB family fusions had higher incidences of alterations in *CTNNB1* (27%, Bonferroni’s post-test, *P* = 0.008) and *NF1* (18%, Bonferroni’s post-test, *P* = 0.09).

Given that multiple oncogenic driver genes were among the top altered genes in our TKI-naive RTK fusion+ cohort, we further assessed the exclusivity and relevance of concomitant driver mutations (Supplementary Table 4). Remarkably, we identified a non-negligible number of concomitant driver mutations. First of all, RTK fusions themselves were not mutually exclusive. We identified three cases that were *ALK* +/*RET*+, two cases with *ALK* +/*ROS1*+, two cases with *RET* +/*ROS1*+, and one case with *ALK* +/*NTRK3*+. In addition, while the functionality of some non-canonical RTK fusions remained to be determined, concomitant driver mutations were detected in many cases with highly recurrent RTK fusions (Supplementary Table 4). Specifically, we identified three lung cancer patients with *EML4*-*ALK*, who each also harbored an activating mutation in *KRAS*, and loss of function mutations in *BRCA2* and *PTEN*, respectively. Six additional lung cancer patients with *KIF5B*-*RET* (*n* = 3), *CCDC6*-*RET* (*n* = 2) or *SDC4*-*ROS1* (*n* = 1) were also detected with activating mutations in *NRAS*, *EGFR*, and *PIK3CA*, as well as loss of function mutations in *BRCA1* and *PTEN*. Moreover, two *FGFR3*-*TACC3*-positive cases also harbored *KRAS-activating* mutations. No concomitant driver mutations in *BRAF* were identified. Of these 18 patients with concomitant drivers, seven (38.9%) harbored these driver mutations in separate subclones, and six had RTK fusion and the other driver co-occurring as clonal events. In the remaining five patients, all RTK fusions were clonal events, in which cases the respective concomitant drivers were subclonal (Supplementary Table 4). In addition, we evaluated the clonality of additional RTK fusions with no detectable co-drivers and found no clear differences in clonality between common recurrent fusions and rare fusion events, other than an increase in the frequency of clonal *RET* fusions in cases with rare fusion partners (Fig. 3c).

### Potential on-target and off-target resistance mechanisms following TKI treatment

**Fig. 4:**
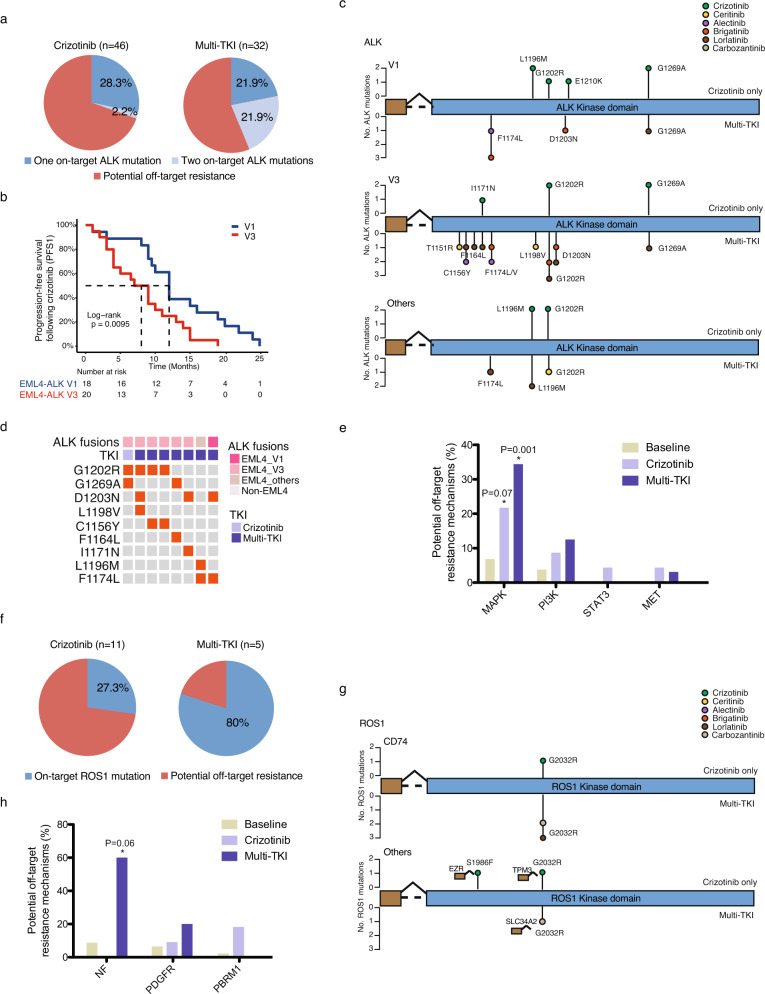
Potential resistance mechanisms in ALK and ROS1 patients following TKI treatment. **a** Proportions of patients carrying on-target *ALK* resistance mutations following ALK TKI treatment. **b** Kaplan–Meier estimates of PFS comparing patients with the two major *EML4*-*ALK* variants following first-line crizotinib. **c** Lollipop plots mapping the on-target resistance mutations in different *ALK* fusion variants following crizotinib or multi-TKI treatments. **d** Resistance mutations in patients who acquired multiple on-target *ALK* mutations following TKI treatment. **e** Potential off-target resistance mechanisms of *ALK* fusions (MAPK pathway included *EGFR*, *NF1*/*2*, *BRAF*, *RAF1*, *KRAS*, and *NRAS* mutations; PI3K pathway included *PIK3CA*, *PTEN*, *AKT2*, and *RICTOR* mutations). **f** Proportions of patients carrying on-target *ROS1* resistance mutations following TKI treatment. **g** Lollipop plots mapping the on-target resistance mutations in different *ROS1* fusion variants following crizotinib or multi-TKI treatments. **h** Potential off-target resistance mechanisms of *ROS1* fusions.

Next, based on evaluable clinical data, we investigated the co-mutational spectrum following targeted TKI treatment aiming to explore the potential resistance mechanisms. In a total of 78 ALK + patients who were treated with ALK TKIs, we detected on-target resistance mechanisms in 30.5% (14/46) and 43.8% (14/32) of patients following crizotinib and multi-TKI treatment, respectively (Fig. 4a and Supplementary Fig. 12A). Moreover, patients treated with multiple TKIs were more likely to acquire more than one on target *ALK* resistance mutations than those treated with crizotinib alone (21.9% vs. 2.2%; Fig. 4a). An increasing trend of RTK fusions following targeted therapies was also observed (Supplementary Fig. 12B), which likely reflects clonal evolution imposed by drug selection. Different *ALK* variants also seemed to respond differently to ALK inhibition. Patients carrying the *EML4*-*ALK* v3 variant had worse progression-free survival (PFS) outcomes compared with those with the v1 variant, both following crizotinib treatment (Hazards ratio (HR) = 2.46, 95%CI = 1.21–4.98, *P* < 0.01; Fig. 4b) and multi-TKI treatment (HR = 2.76, 95%CI = 0.71–10.72, *P* = 0.13; Supplementary Fig. 13A). Differences in PFS outcome might be attributed to a more complex spectrum of on-target resistance (Fig. 4c), as well as a higher incidence of acquiring multiple resistance mutations following TKI treatment (Fig. 4d) in the V3 variant. On the other hand, *ALK* + patients with other *EML4*-*ALK* variants or non-*EML4* partners, including previously unreported *MEMO1*-*ALK* and *WRD43*-*ALK* fusions, also responded well to first-line crizotinib treatment, with a median PFS of 11 months (Supplementary Fig. 13B). In addition, there were significant increases in MAPK pathway gene alterations (crizotinib, FDR-adjusted *P* = 0.07; multi-TKI, FDR-adjusted *P* = 0.001), as well as higher frequencies of PI3K pathway, *STAT3* and *MET* alterations following ALK inhibition (Fig. 4e), which might be associated with off-target TKI resistance.

Similarly, in *ROS1* + patients, on-target *ROS1* resistance mutations were identified in 27.3% (3/11) and 80% (4/5) of cases treated with crizotinib and multi-TKI, respectively (Fig. 4f). The most common on-target resistance mutations in this study cohort was p.G2032R, which could be induced by both first-line crizotinib and second- or subsequent-line TKIs (Fig. 4g). Finally, comparisons of TKI-naive and -treated patients revealed increases in *NF1*/*2* (FDR-adjusted *P* = 0.06), *PDGFR* and *PBRM1* alterations following TKI resistance (Fig. 4h).

In addition to the top altered fusion genes, we also identified a number of cases with relatively uncommon fusions that were treated with TKI therapies. In particular, we identified two lung cancer cases, one (PT218) with an *HLA-DRB1*-*MET* fusion gene and the other (PT230) with an *IRF2BP2*-*NTRK1* fusion gene. Both fusion genes were clonal mutations and no additional known oncogenic mutations were identified, suggesting that the fusion genes were the sole driving events in these tumors. Targeted RNA sequencing confirmed the expression of the *HLA*-*DRB1*-*MET* fusion (Supplementary Fig. 14). Following crizotinib treatment, the *MET* + patient remained disease-free for 6 months, and the *NTRK1* + patient reached a remarkable PFS of 18.5 months.

## Discussion

Understanding of recurrent genetic fusions, particularly those of RTKs, may facilitate drug development. An increasing number of TKIs have been developed to target RTK gene aberrations and provide the most promising therapeutic effects for cancer patients. In this study, we described the RTK fusion events in 1324 patients across diverse solid tumors using DNA-based next-generation sequencing profiling. We were able to characterize the prevalence and genomic structures of RTK fusions. While a number of cancer types showed a wide spectrum of RTK fusions, certain cancer types, such as thyroid cancer, urological cancers, and neuroendocrine tumors, are very selective in the RTK fusion genes they carry. The association between these RTK fusion genes and the cancer type makes them highly valuable for diagnostic purposes. In addition, we found that except for the highly reported fusion genes, such as *EML4*-*ALK*, *KIF5B*-*RET*, *CD74*-*ROS1*, and *FGFR3*-*TACC3*, the majority of fusion events were largely non-recurrent. Nearly all RTK genes, with the exception of *FGFR3*, were highly promiscuous in their fusion partner, which might be scattered across the genome. Our study also suggested that different RTK fusions and their respective variants might display varying spectra of concomitant and acquired resistance mutations prior to and following TKI treatments, respectively, and consequently impact the patients’ response to TKIs. The above findings highlight the importance of screening using high-throughput sequencing technologies.

Although RTK fusions had largely been considered to occur mutually exclusively to other oncogenic driver mutations, several studies have provided evidence supporting the co-existence of dual drivers^15,16^. In addition, patients with dual drivers may exhibit variable response to single-agent targeted therapies. The best-studied example is concomitant *EGFR-*activating mutations and *ALK* fusions. However, in such patients, it remains unclear whether they responded equally well to single-agent TKIs or combination TKI therapies are needed for prolonged survival benefit^15–20^. The choice of therapy may depend on the relative abundance or activation levels of the two drivers^16,18^. Taking advantage of high-throughput sequencing technologies, we identified several cases (~7% of RTK fusion+ baseline samples) with dual driver alterations. Concomitant driver mutations included activating mutations in classic oncogenes (e.g., *KRAS*, *NRAS*, *EGFR*, and *PIK3CA*), as well as loss-of-function mutations in tumor suppressors (e.g., *BRCA1*/*2* and *PTEN*). Notably, such driver events co-existed with the RTK fusion either in the same clone or as distinct subclones, which might further influence disease progression and treatment outcome. In addition, we showed that different RTKs varied in their repertoire of concomitant driver and other somatic mutations, and may serve as potential intrinsic resistance mechanisms to targeted therapies. However, due to the retrospective nature of our study, which has inadequate clinical follow-up data, further investigations are necessary to dissect the effect of concurrent alterations on clinical response to RTK fusion-targeted inhibitors.

As mentioned, our study reported a considerable number of rare RTK fusions; some of which have previously been reported in sporadic cases, for other uncommon fusions, their driver roles might require additional confirmation in the absence of treatment outcomes. Despite the mostly non-recurrent nature of RTK fusions, numerous studies, including those of our own, have shown that patients with rare fusion events can be successfully targeted by TKI treatment. For example, favorable responses to TKI therapies have been demonstrated against the less common ALK fusion genes, such as *STRN*-*ALK*, *CUX1*-*ALK*, and *GCC2*-*ALK*^21–23^. In line with previous reports, we showed that patients with non-v1/v3 variants of *ALK*, including previously unreported *ALK* fusions, might also derive long-term clinical benefit from TKI treatment. Similarly, we also reported two relatively uncommon *MET* and *NTRK1* fusions, against which crizotinib was shown to be effective. These results might support the use of DNA-based sequencing strategies for fusion screening to inform clinical actions.

It is worth noting that while both DNA- and RNA-based sequencing approaches are commonly used in the research and diagnostic settings, each has their own unique advantages and disadvantages. DNA-based approach is more clinically applicable and allows for the detection of ctDNA using liquid biopsies. Although “hotspot” breakpoints and common partner genes exist for most RTK fusions, whole genome-based sequencing approach does not rely on the design and performance of targeted panels and would enable an even coverage of all potential structural alterations, particularly those that occur in the intronic regions. By contrast, targeted approach is more cost-effective and offers a greater sequencing depth, and consequently higher sensitivity at regions of strong clinical relevance. On the other hand, RNA-based approach would depend on the quality of the sample but has a unique advantage in detecting functional fusion events as compared to DNA-based methods. For example, it has been shown that a considerable portion of rare or IGR fusion events as detected by DNA-based sequencing approach are common fusion genes at the RNA level^24,25^. In our study, we confirmed our DNA-based finding in a case with a rare *HLA*-*DRB1*-*MET* fusion who had responded to crizotinib by using targeted RNA sequencing. The limitations and challenges of DNA- and RNA-based sequencing approaches can likely be overcome by a complementary approach combining the two methods.

In addition to rare fusion genes, different fusion variants may also influence clinical response or the development of resistance to TKI therapies. In line with studies on *ALK*-positive lung cancers, which have showed the variable clinical outcome of patients with different *EML4*-*ALK* fusion variants^26,27^, we also observed prolonged PFS outcome in patients harboring the v1 variant compared with those carrying the v3 variant. Interestingly, we found that the v3 variant were more likely to acquire two or more on-target resistance mutations than the v1 variant. In addition, the spectrum of resistance mutations in the v3 variant was more complex. The difference in their ability to acquire on-target resistance mutations might explain the preferential clinical outcome associated with the v1 variant.

Numerous preclinical and clinical studies have led to the rapid expansion of first- and next-generation TKIs for aberrations in RTKs. Given the functional conservation of RTKs, many of which can be targeted by multi-kinase inhibitors with activity against various targets. For example, crizotinib has demonstrated activity across a wide range of targets, including *ALK*, *RET*, *ROS1*, *NTRK*, and *MET*, in our study cohort. However, the activities of multi-kinase inhibitors can vary widely based on their selectivity for the targets of interest. Recent advances in the highly selective TKIs have largely increased the proportion of patients that can benefit from TKI therapies. NTRK inhibitors, including larotrectinib and entrectinib, represent the best examples that are associated with remarkable response rates (>75%) on *NTRK*-positive tumors regardless of cancer types^28–30^. Next-generation NTRK inhibitors have shown promising results in overcoming acquired resistance to first-generation inhibitors, and are currently undergoing clinical development^31,32^. Other examples of selective RTK inhibitors include those against RET protein, BLU-667 (pralsetinib) and LOXO-292 (selpercatinib), which also demonstrated potent activity against *RET* fusions and activating mutations across a multitude of cancer types^33–35^.

In addition, FGFR inhibitors are being rapidly developed in the clinic. Erdafitinib, an oral pan-FGFR inhibitor, was the first *FGFR*-selective compound approved by FDA for the second-line treatment of metastatic urothelial carcinoma with an *FGFR2* or *FGFR3* alteration^36^. More recently, pemigatinib was approved for previously treated cholangiocarcinoma with *FGFR2* fusions or rearrangements^37^, with FoundationOne®CDx as the companion diagnostics. Our study also supported the use of NGS to screen for genetic fusion events. In the TCGA cohort of 285 cancer patients that underwent comprehensive RNA-seq^5^, only one patient (0.35%) was found to carry a *FGFR2* fusion. Similarly in our multi-cancer cohort, we identified a total of 89 *FGFR* fusion-positive patients, accounting for 0.40% of the cancer patients overall.

Taken together, our study described the landscape of RTK fusions and their associated mutational spectrum in a large cohort of cancer patients with diverse cancers. The largely non-recurrent and complex nature of RTK fusions, the existence of concomitant somatic mutations, together with the differences in acquired resistance mutations among different fusion variants all emphasize the need for high-throughput sequencing technologies to fully capture fusion events and better inform clinical diagnosis and treatment strategies. Our findings support the diagnostic and prognostic values of RTK fusions and highlight the importance of RTK fusion screening in relevant cancer types and future clinical trials to facilitate the clinical development of therapeutic strategies to target these aberrations.

## Methods

### Study cohort and sample collection

The study retrospectively reviewed the clinico-genomics database of Geneseeq Technology Inc., China consisting of cancer patients who were routinely treated at multiple hospitals, including the Third Affiliated Hospital of Sun Yat-sen University and the National Cancer Center/National Clinical Research Center for Cancer/Cancer Hospital, between August 2015 and January 2020. Targeted genomic sequencing which encompasses all exons and flanking intronic regions of the reported RTKs, as well as selected exon and intronic regions of the respective previously reported fusion partners, was performed on the tumor specimen and/or circulating cell-free DNA (cfDNA) from body fluids, including plasma, pleural effusion and cerebrospinal fluid. Sample processing and sequencing were performed in a CLIA-certified and CAP-accredited laboratory (Geneseeq Technology Inc., Nanjing, China). Patient information was retrospectively reviewed. For patients with sufficient clinical follow-up data, progression-free survival (PFS) was defined as the time from the beginning of TKI treatment to the date of progressive disease. PFS2 was defined as the time from the beginning of the respective second-line TKI treatment to the date of disease progression. Informed written consent was obtained from each subject or the subject’s family member upon sample collection according to the protocols approved by the ethics committee of each hospital.

### Next-generation sequencing

Next-generation sequencing (NGS) was performed as previously described^38^. In brief, genomic DNAs from tissue or circulating cell-free DNA from body fluids were extracted. Customized xGen lockdown probes (Integrated DNA Technologies) targeting 425 cancer-relevant genes were used for hybridization enrichment. Libraries were on-beads PCR-amplified, purified, sized and quantified, and sequenced on an Illumina HiSeq4000 platform. The mean coverage depth was 143X for controls, 1341X for tissues, and 4185X for cfDNA samples.

### Mutation calling

Trimmomatic^39^ was used for FASTQ file quality control. Leading/trailing low quality (quality reading below 20) or N bases were removed. Paired-end reads were then aligned to the reference human genome (build hg19), using the Burrows-Wheeler Aligner^40^ with the default parameters. PCR deduplication was performed using Picard and local realignment around indels and base quality score recalibration were performed using GATK3^41^. Further, samples with mean dedup depth <30X were removed. Single nucleotide variants (SNVs) and indels were identified using VarScan2^42^, with a minimum variant allele frequency threshold set at 0.01 and *p*-value threshold for calling variants set at 0.05 to generate Variant Call Format files. All SNVs/indels were annotated with ANNOVAR, and each SNV/indel was manually checked on the Integrative Genomics Viewer. Variants were further filtered with the following parameters: (i) minimum read depth = 20; (ii) minimum base quality = 15; (iii) minimum variant supporting reads = 5; (iv) variant supporting reads mapped to both strands; (v) strand bias no greater than 10%; (vi) if present in >1% population frequency in the 1000 g or ExAC database and vii) through an internally collected list of recurrent sequencing errors using a normal pool of 100 samples. The sequencing assay has been validated in compliance with CAP and CLIA with a limit of detection of 1% VAF. Copy number variations (CNVs) were called by FACETS^43^ (Fraction and Allele-Specific Copy Number Estimates from Tumor Sequencing) to obtain tumor purity-, ploidy-, and clonal heterogeneity-adjusted copy number data. Fusion events were called using the Delly fusion callying tool^44^ to identify the number of chimeric reads (sequencing paired ends mapped to different genes) and split reads (spanning a fusion breakpoint) from the targeted DNA-seq data. RTK fusions were filtered if (i) split reads <3 or paired reads <5, or (ii) lack of an intact kinase domain. All fusions were manually confirmed using the Integrative Genomics Viewer (IGV).

### Clonality analysis

To infer the clonality of concomitant driver mutations, we used Pyclone^45^ to estimate the cancer cell fraction (CCF) of each mutation, with CCF > 0.6 considered as clonal mutations and CCF ≤ 0.6 considered as subclonal. For fusion events, CCF values were converted from the estimation of variant allele frequency of each fusion gene.

### Statistical analysis

Comparisons of proportion between groups were done using the Fisher’s exact test. A two-sided *p* value <0.05 was considered significant. *P* values were adjusted for multiple group comparisons by Bonferonni’s post hoc test or corrected for multiple hypotheses testing using the false discovery rate (FDR) adjustment method as appropriate. Adjusted *p* value <0.1 was considered significant. For survival analyses, Kaplan–Meier curves were compared using the log-rank test, and hazard ratios (HRs) were calculated by Cox proportional hazards model. All statistical analyses were done in R (v.3.5.2).

### Reporting summary

Further information on research design is available in the Nature Research Reporting Summary linked to this article.

## Supplementary information

Supplementary figures and tables

REPORTING SUMMARY

## Data Availability

The data that support the findings of this study are available from the corresponding authors upon reasonable request. The raw sequencing data are available from GSA for human using the accession code HRA003240.
