# Supplementary material for: Landscape of potentially targetable receptor tyrosine kinase fusions in diverse cancers by DNA-based profiling
Source: NPJ Precis Oncol. 2022 Nov 11;6:84. doi: 10.1038/s41698-022-00325-0 (PMC9652465; doi:10.1038/s41698-022-00325-0)
Supplement: Supplementary file 1 — Supplementary figures and tables [file 41698_2022_325_MOESM1_ESM.pdf]

Supplementary Figure 1

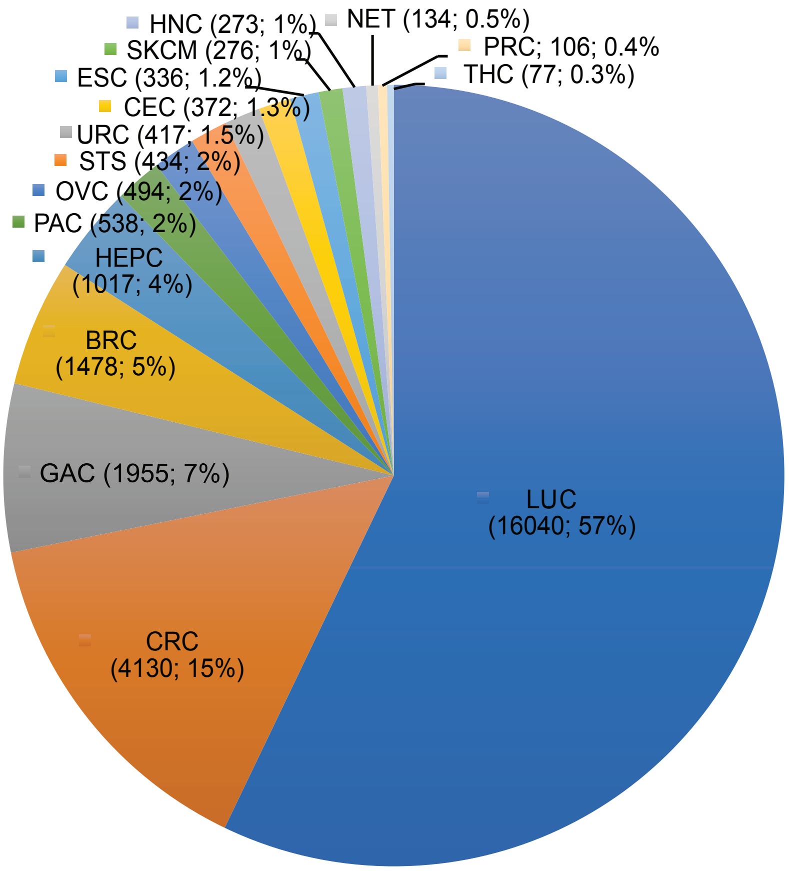

Supplementary Figure 1. Distribution of cancer types in the study cohort.
LUC, lung cancer; CRC, colorectal cancer; GAC, gastric cancer; BRC, breast cancer; HEPC, hepatobiliary cancer; PAC, pancreatic cancer; OVC, ovarian cancer; STS, soft tissue sarcoma; CEC, cervical cancer; ESC, esophageal cancer; URC, urinary cancer; HNC, head and neck cancer; SKCM, skin cutaneous melanoma; Net, neuroendocrine tumor; PRC, prostate cancer; THC, thyroid cancer

Supplementary Figure 2

A

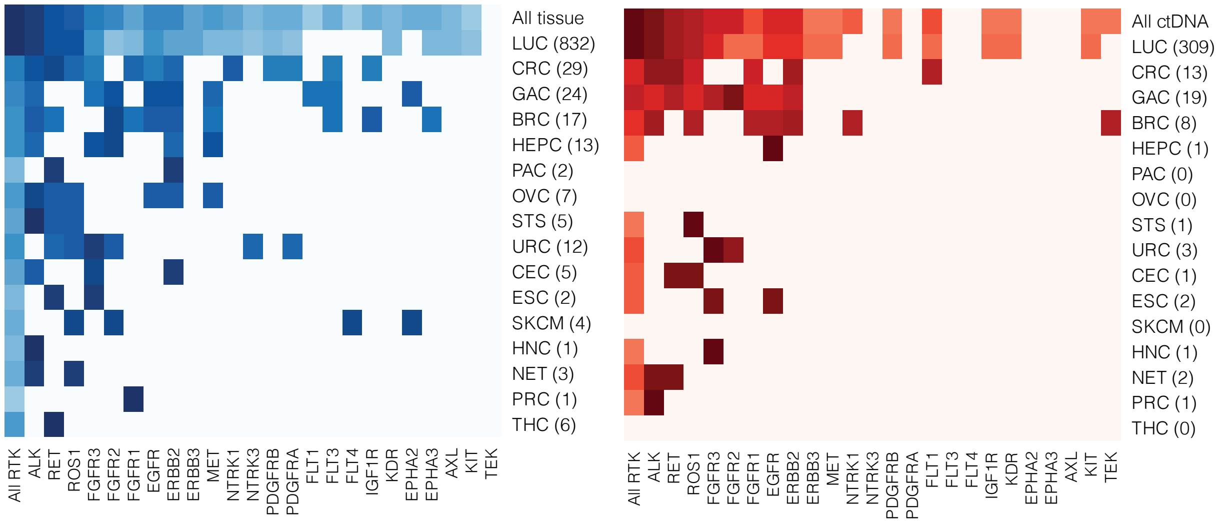

B

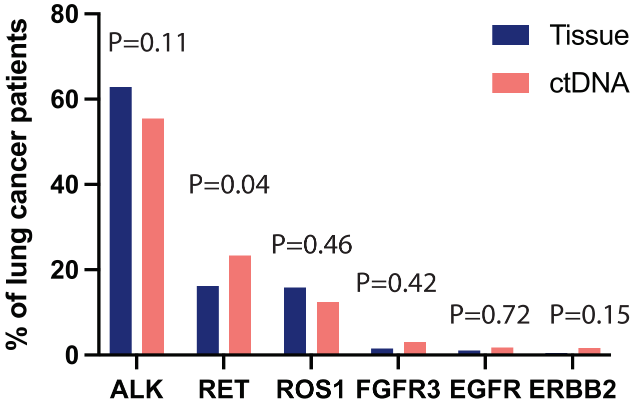

Supplementary Figure 2. Detection of RTK fusions in tumor and cfDNA specimens.
A, Heatmaps showing the prevalence of RTK fusions in different cancers comparing tumor specimens and ctDNA samples. B, Bar graph showing similar RTK fusion frequencies between the sample origins, except for a higher prevalence of RET fusion in the ctDNA. The FDR adjusted q values were indicated.

Supplementary Figure 3

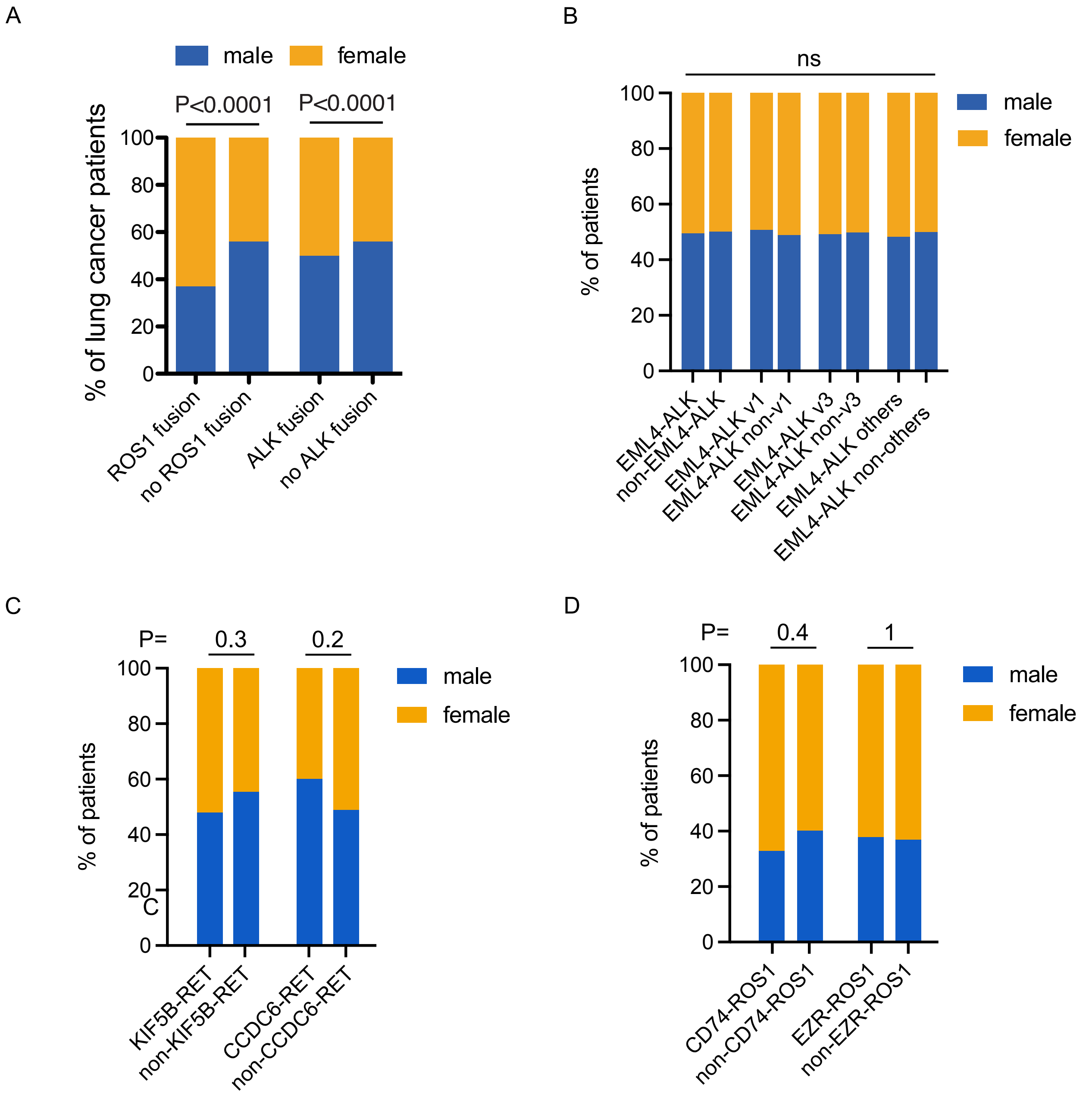

Supplementary Figure 3. Associations of different fusion events with patient sex.
A, ROS1 and ALK fusions showed increased associations with the female sex in the lung cancer cohort. B-D, No associations with sex among different fusion variants of (B) ALK, (C) RET, or (D) ROS1.

Supplementary Figure 4

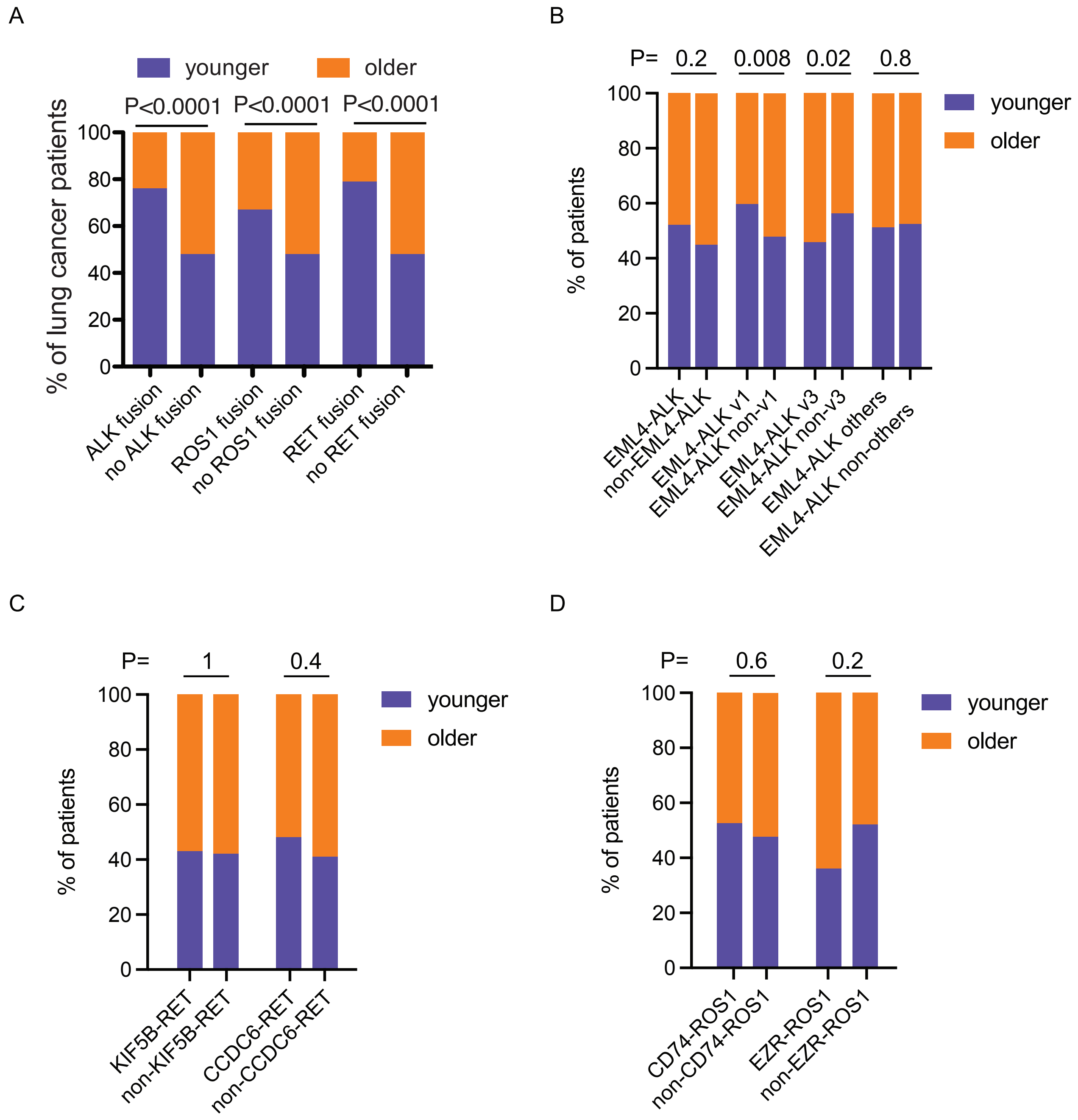

Supplementary Figure 4. Associations of different fusion events with patient age.
A, ALK, ROS1 and RET fusions showed increased associations with earlier disease onset in the lung cancer cohort. B, Associations of the ALK v1 variant with younger age and the v3 variant with older age. C-D, No associations with age among different fusion variants of (C) RET or (D) ROS1.

Supplementary Figure 5

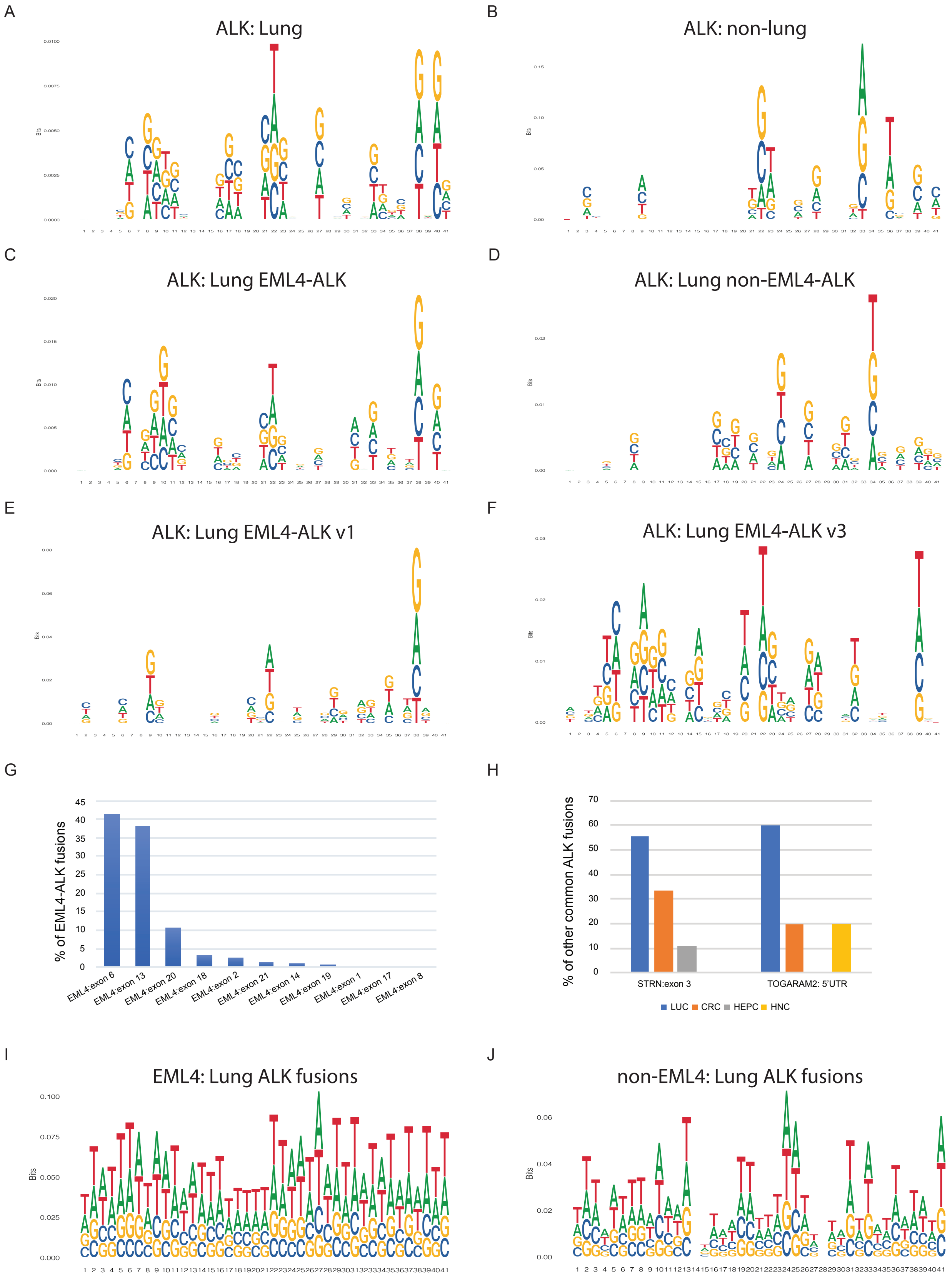

Supplementary Figure 5. Genomic structures of ALK fusions.

A-F, consensus plots showing the sequencing patterns ±20 bp around the breakpoints of the ALK gene in (A) lung cancer, (B) non-lung cancer, (C) EML4-ALK, (D) non-EML4-ALK, (E) EML4-ALK v1 variant, and (F) EML4-ALK v3 variant. G-H, Frequencies of breakpoints in (G) EML4 and (H) other common ALK fusion partners. I-J , consensus plots showing the sequencing patterns ±20 bp around the breakpoints of (I) EML4 and (J) non-EML4 ALK partners.

Supplementary Figure 6

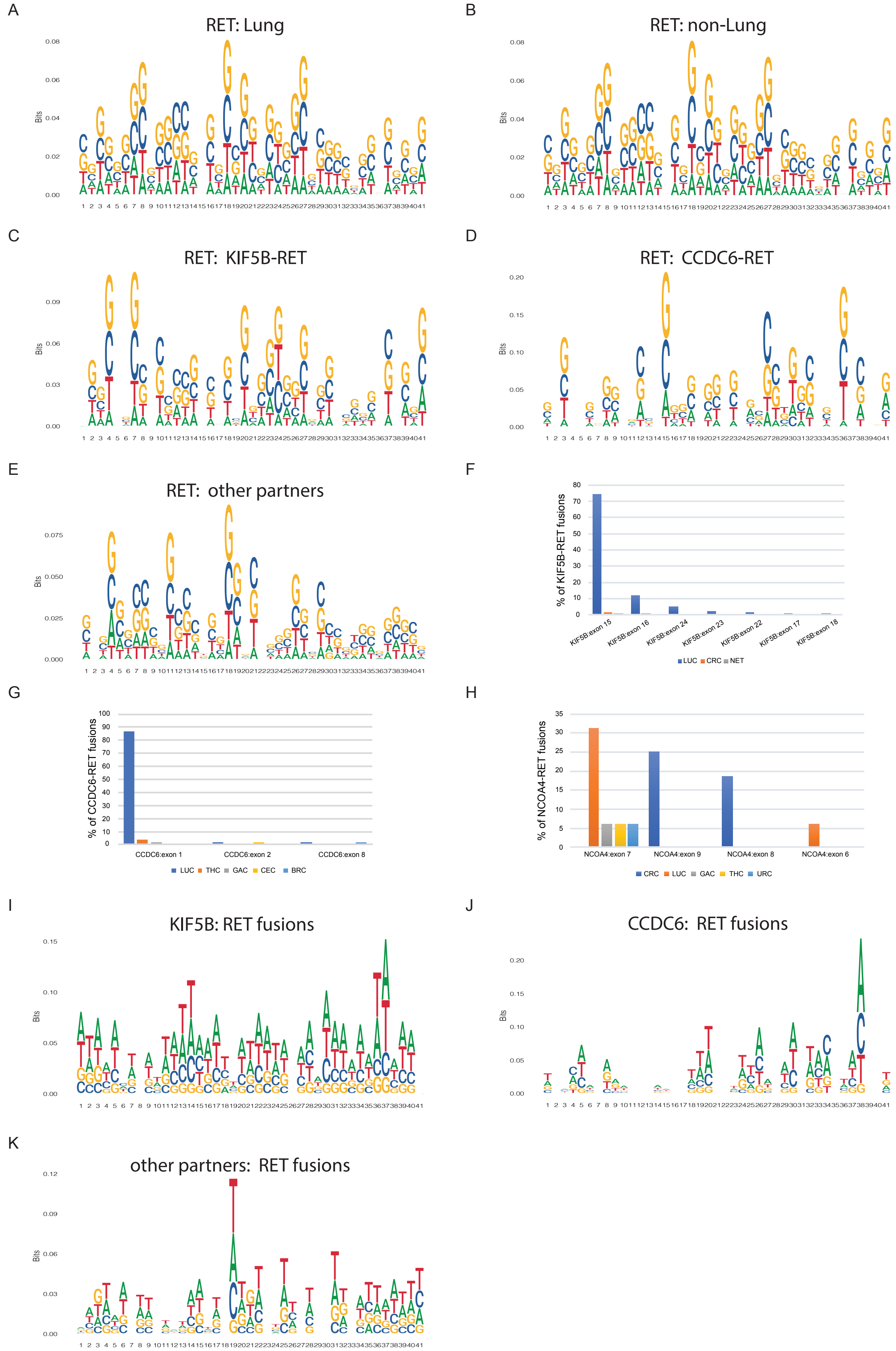

Supplementary Figure 6. Genomic structures of RET fusions.
A-E, consensus plots showing the sequencing patterns ±20 bp around the breakpoints of the RET gene in (A) lung cancer, (B) non-lung cancer, (C) KIF5B-RET, (D) CCDC6-RET, and (E) all other RET fusions. F-H, Frequencies of breakpoints in (F) KIF5B, (G) CCDC6, and (H) NCOA4. I-K, consensus plots showing the sequencing patterns ±20 bp around the breakpoints of (I) KIF5B, (J) CCDC6, and (K) all other RET fusion partners.

Supplementary Figure 7

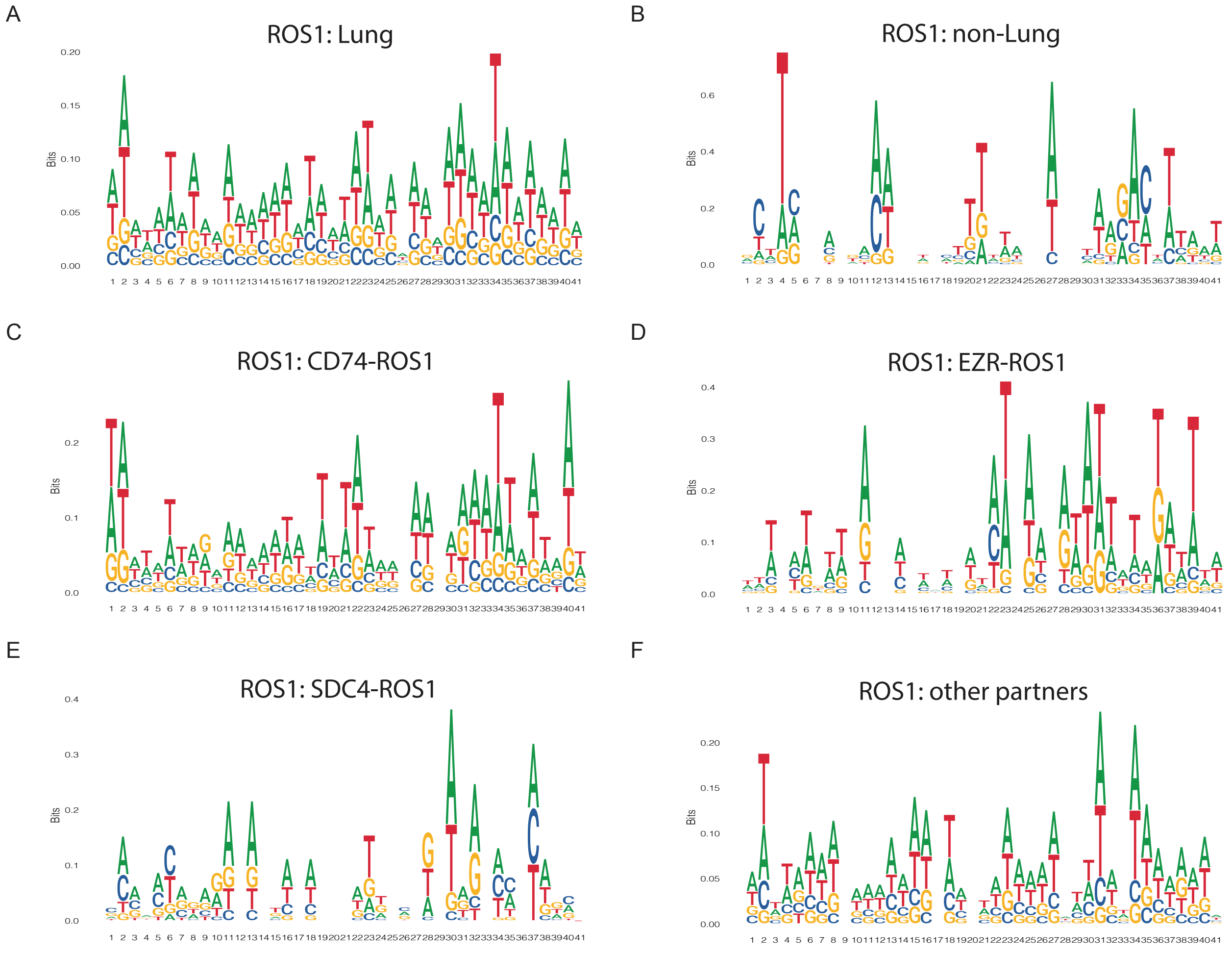

Supplementary Figure 7. Consensus plots of ROS1 breakpoints.
A-F, consensus plots showing the sequencing patterns ±20 bp around the breakpoints of the ROS1 gene in (A) lung cancer, (B) non-lung cancer, (C) CD74-ROS1, (D) EZR-ROS1, (E) SDC4-ROS1, and (F) other ROS1 fusions.

Supplementary Figure 8

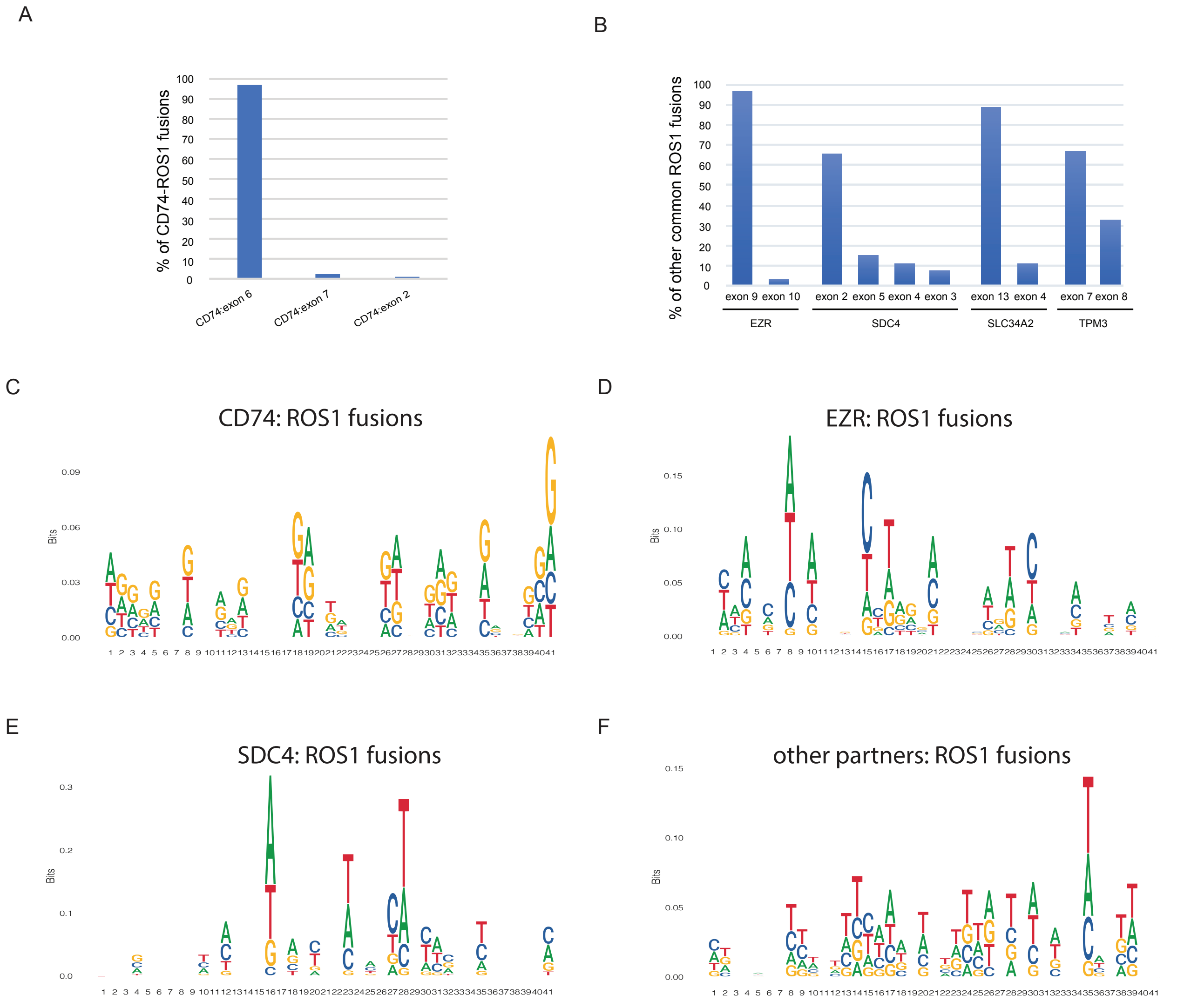

Supplementary Figure 8. Breakpoint structures of ROS1 fusion partners.
A-B, Frequencies of breakpoints in (A) CD74, (B) other common ROS1 fusion partners. C-F, consensus plots showing the sequencing patterns ±20 bp around the breakpoints of (C) CD74, (D) EZR, (E) SDC4, and (F) all other ROS1 fusion partners.

Supplementary Figure 9

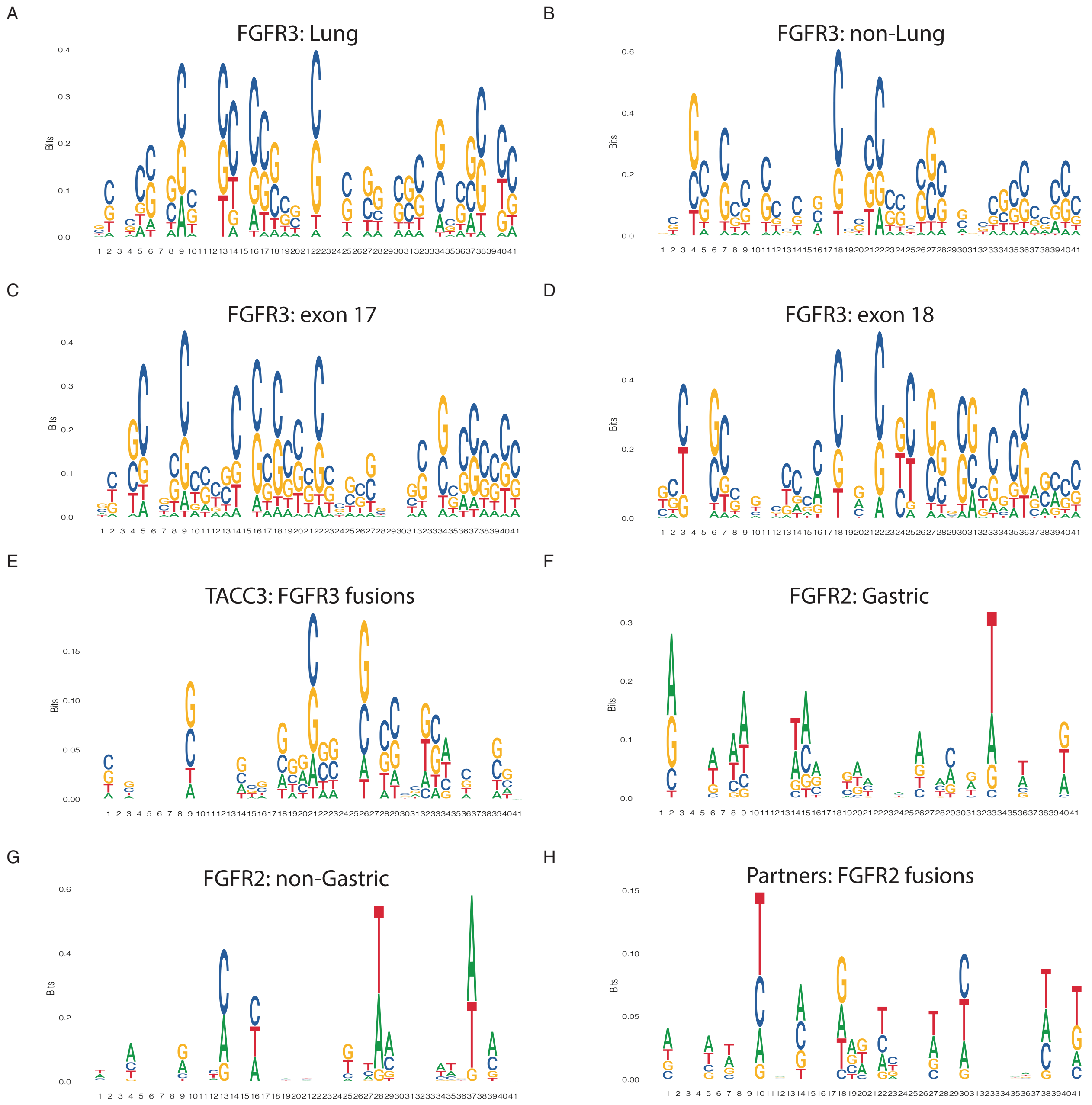

Supplementary Figure 9. Consensus plots of FGFR family breakpoints.
A-D, consensus plots showing the sequencing patterns ±20 bp around the breakpoints of FGFR3 in (A) lung cancer, (B) non-lung cancer, and cases with (C) exon 17 and (D) exon 18 fusions. E, consensus plots showing the sequencing patterns ±20 bp around the breakpoints of TACC3. F-G, consensus plots showing the sequencing patterns ±20 bp around the breakpoints of FGFR2 in (F) gastric cancer and (G) non-gastric cancers. H, consensus plots showing the sequencing patterns ±20 bp around the breakpoints of FGFR2 fusion partners.

Supplementary Figure 10

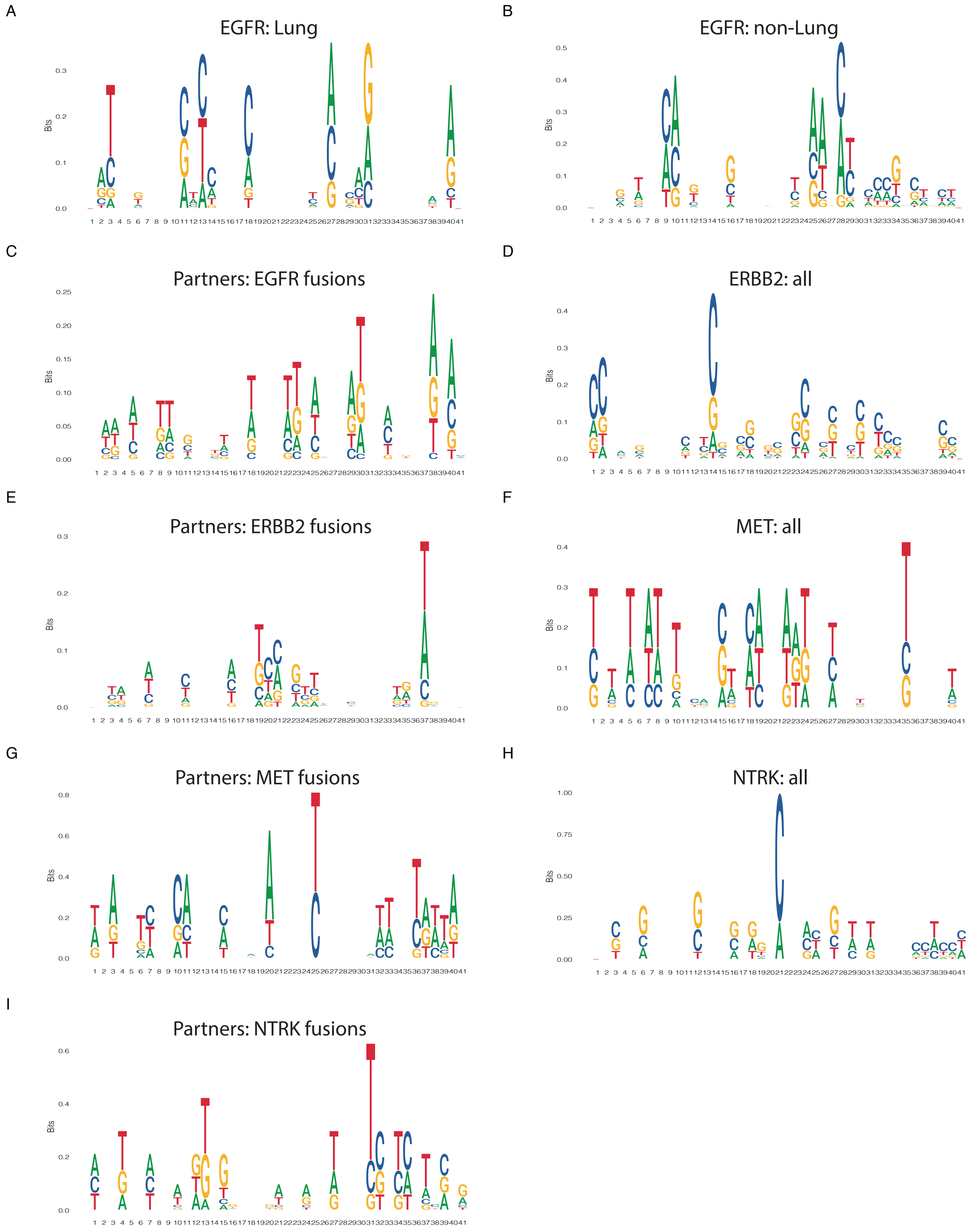

Supplementary Figure 10. Breakpoint structures of all other common fusions.
A-I, consensus plots showing the sequencing patterns ±20 bp around the breakpoints of (A) EGFR in lung cancer, (B) EGFR in non-lung cancer, (C) EGFR fusion partners, (D) ERBB2, (E) ERBB2 fusion partners, (F) MET, (G) MET fusion partners, (H) NTRK and (I) NTRK fusion partners.

Supplementary Figure 11

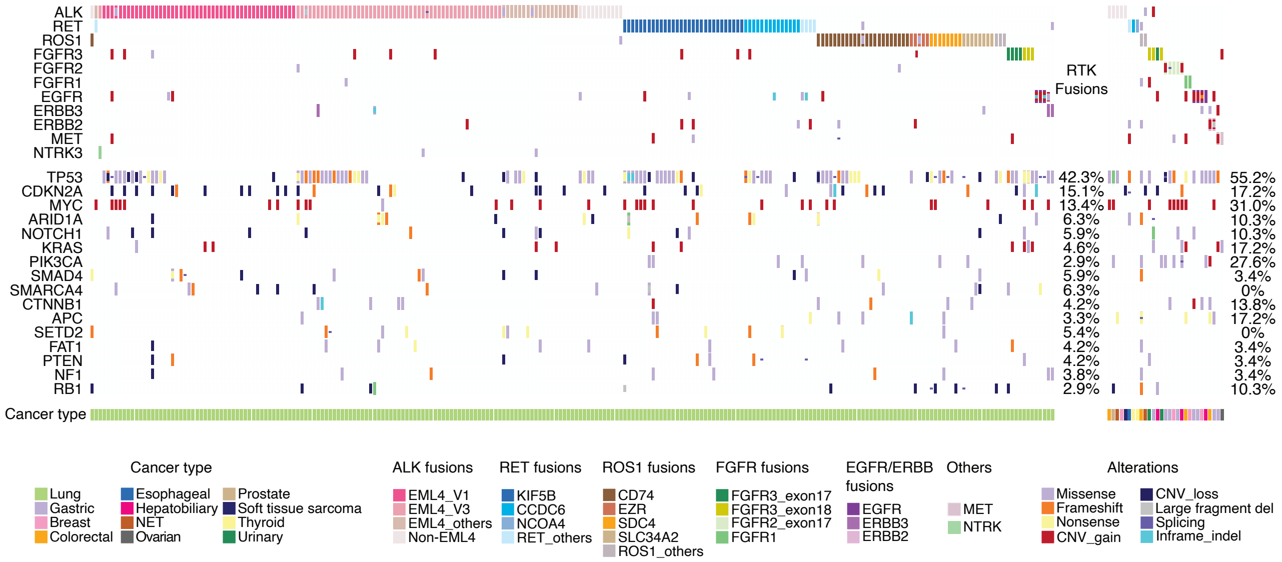

Supplementary Figure 11. Mutational landscape of concomitant mutations comparing lung and non-lung cancers prior to TKI treatment.

Supplementary Figure 12

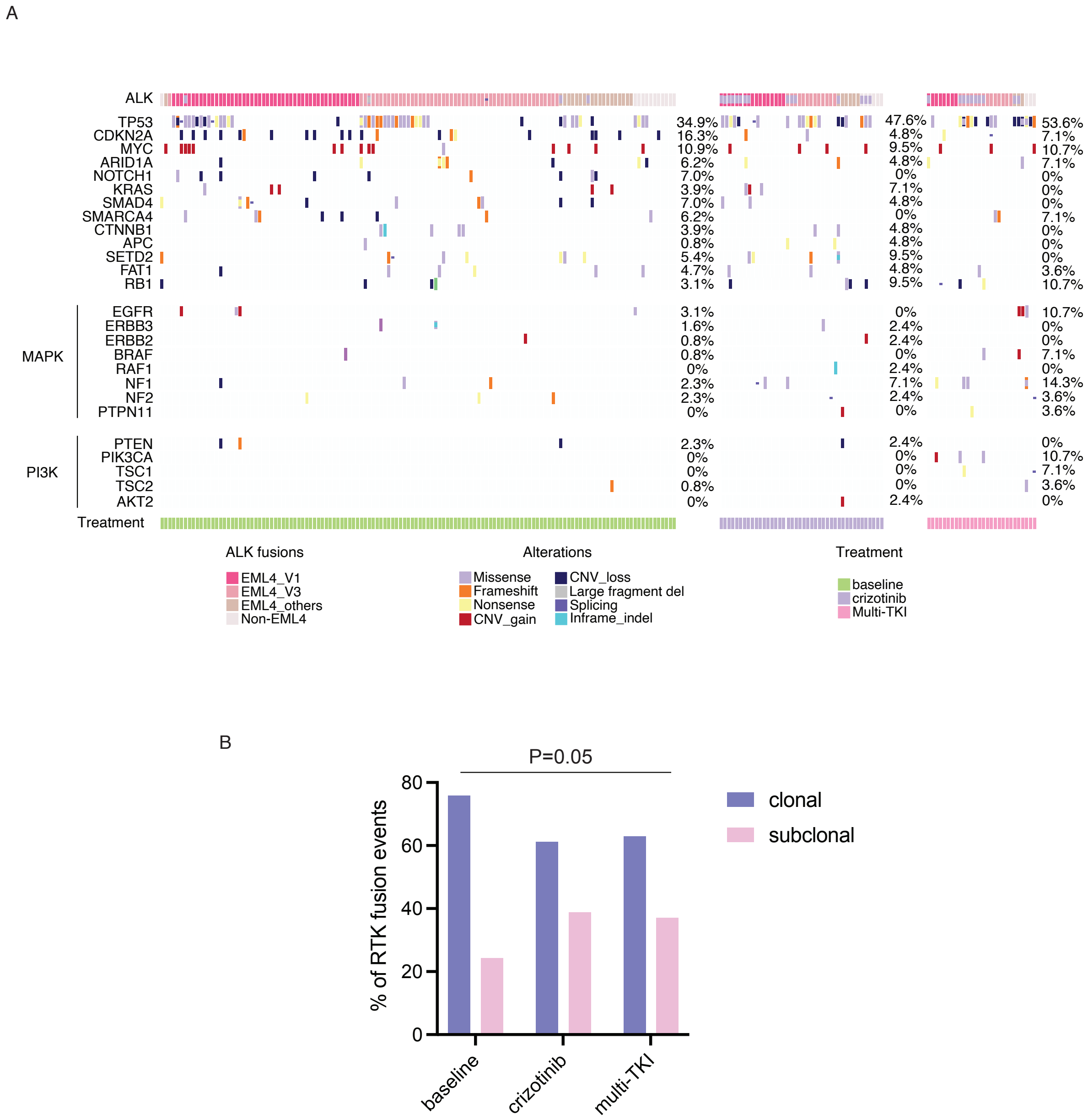

Supplementary Figure 12. Resistance to RTK fusion-targeted therapies
A, mutational landscape of ALK fusion-positive cases pre- and post-TKI treatment. B, An increasing trend towards a higher frequency of subclonal RTK fusions following treatment.

Supplementary Figure 13

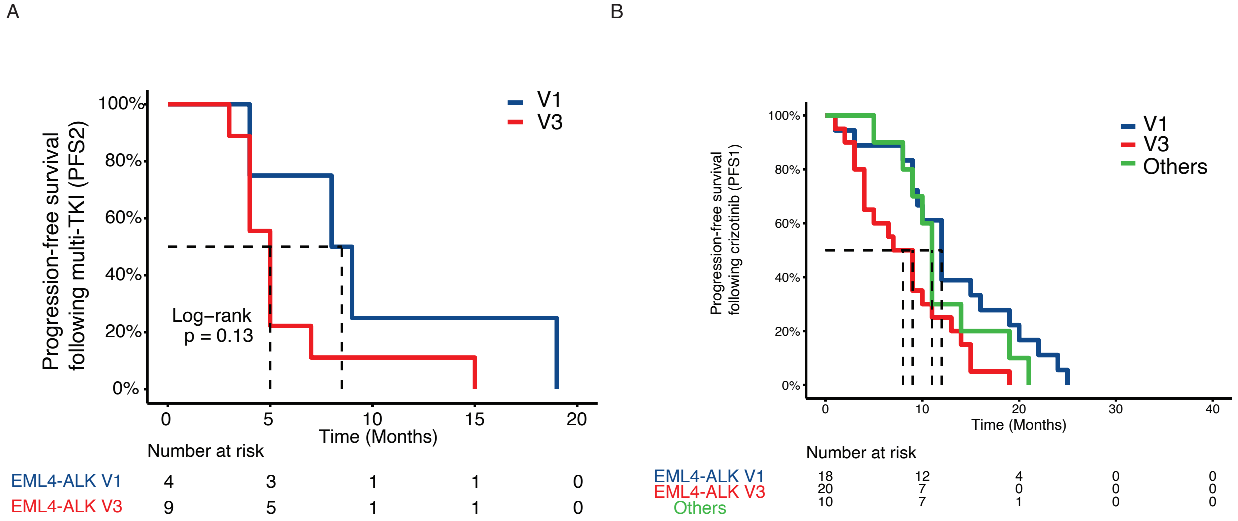

Supplementary Figure 13. Kaplan-Meier estimates of PFS comparing different ALK fusion variants following TKI treatment.
A, Kaplan-Meier estimates of PFS comparing patients with the two major ALK fusion variants following multi-TKI treatment. B, Kaplan-Meier estimates of PFS comparing patients with different ALK fusion variants following crizotinib treatment.

Supplementary Figure 14

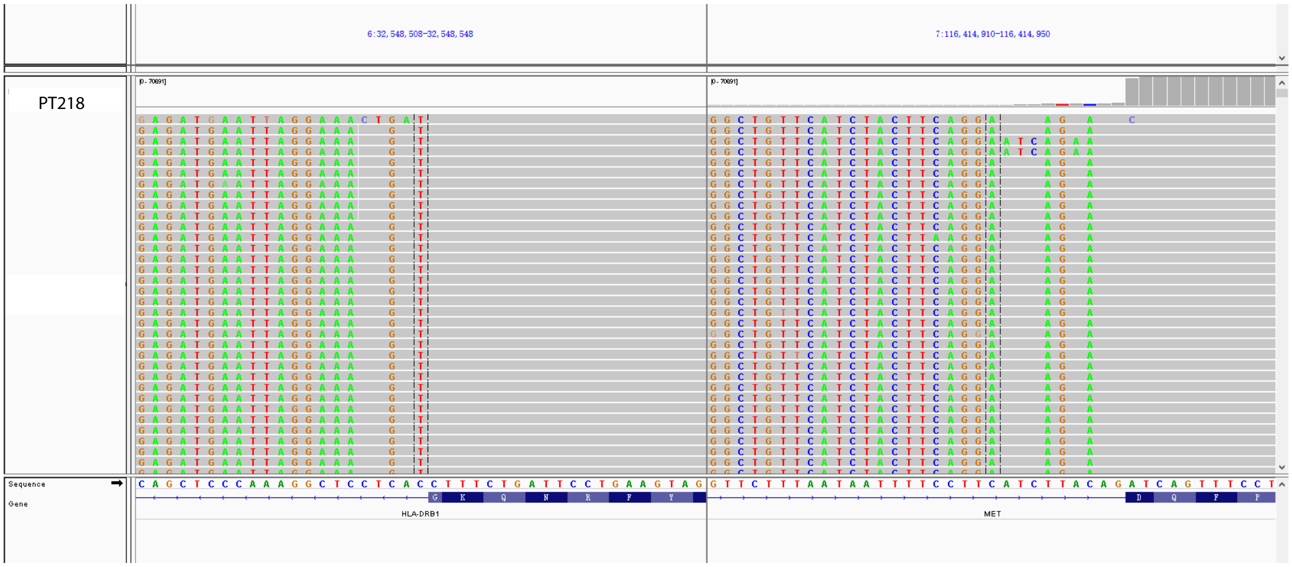

Supplementary Figure 14. IGV showing the breakpoints of the HLA-DRB1-MET fusion.

Supplementary Table 1. Overall frequencies of RTK fusions across different cancer types.

	all RTK	ALK	RET	ROS1	FGFR3	FGFR2	FGFR1	EGFR	ERBB2	ERBB3	MET	NTRK1	NTRK3	PDGFRB	PDGFRA	FLT1	FLT3	FLT4	IGF1R	KDR	EPHA2	EPHA3	AXL	KIT	TEK
THC (77)	7.79	0.00	7.79	0.00	0.00	0.00	0.00	0.00	0.00	0.00	0.00	0.00	0.00	0.00	0.00	0.00	0.00	0.00	0.00	0.00	0.00	0.00	0.00	0.00	0.00
PRC (106)	1.89	0.94	0.00	0.00	0.00	0.00	0.94	0.00	0.00	0.00	0.00	0.00	0.00	0.00	0.00	0.00	0.00	0.00	0.00	0.00	0.00	0.00	0.00	0.00	0.00
NET (134)	3.73	2.24	0.75	0.75	0.00	0.00	0.00	0.00	0.00	0.00	0.00	0.00	0.00	0.00	0.00	0.00	0.00	0.00	0.00	0.00	0.00	0.00	0.00	0.00	0.00
HNC (273)	0.73	0.37	0.00	0.00	0.37	0.00	0.00	0.00	0.00	0.00	0.00	0.00	0.00	0.00	0.00	0.00	0.00	0.00	0.00	0.00	0.00	0.00	0.00	0.00	0.00
SKCM (276)	1.45	0.00	0.00	0.36	0.00	0.36	0.00	0.00	0.00	0.00	0.00	0.00	0.00	0.00	0.00	0.00	0.00	0.36	0.00	0.00	0.36	0.00	0.00	0.00	0.00
ESC (336)	1.19	0.00	0.30	0.00	0.60	0.00	0.00	0.30	0.00	0.00	0.00	0.00	0.00	0.00	0.00	0.00	0.00	0.00	0.00	0.00	0.00	0.00	0.00	0.00	0.00
CEC (372)	1.61	0.27	0.27	0.27	0.54	0.00	0.00	0.00	1.08	0.27	0.00	0.00	0.00	0.00	0.00	0.00	0.00	0.00	0.00	0.00	0.00	0.00	0.00	0.00	0.00
URC (417)	3.60	0.00	0.24	0.24	2.16	0.72	0.00	0.00	0.00	0.00	0.00	0.00	0.24	0.00	0.24	0.00	0.00	0.00	0.00	0.00	0.00	0.00	0.00	0.00	0.00
STS (434)	1.38	0.69	0.23	0.46	0.00	0.00	0.00	0.00	0.00	0.00	0.00	0.00	0.00	0.00	0.00	0.00	0.00	0.00	0.00	0.00	0.00	0.00	0.00	0.00	0.00
OVC (494)	1.62	0.61	0.20	0.40	0.00	0.00	0.00	0.20	0.20	0.00	0.20	0.00	0.00	0.00	0.00	0.00	0.00	0.00	0.00	0.00	0.00	0.00	0.00	0.00	0.00
PAC (538)	0.37	0.00	0.19	0.00	0.00	0.00	0.00	0.00	0.19	0.00	0.00	0.00	0.00	0.00	0.00	0.00	0.00	0.00	0.00	0.00	0.00	0.00	0.00	0.00	0.00
HEPC (1,017)	1.38	0.10	0.00	0.00	0.29	0.49	0.00	0.20	0.10	0.00	0.29	0.00	0.00	0.00	0.00	0.00	0.00	0.00	0.00	0.00	0.00	0.00	0.00	0.00	0.00
BRC (1,478)	1.69	0.27	0.07	0.07	0.00	0.34	0.14	0.20	0.27	0.00	0.07	0.14	0.00	0.00	0.00	0.00	0.07	0.00	0.14	0.00	0.00	0.07	0.00	0.00	0.07
GAC (1,955)	2.20	0.15	0.10	0.05	0.20	1.18	0.05	0.26	0.41	0.00	0.10	0.00	0.00	0.00	0.00	0.05	0.05	0.00	0.00	0.00	0.15	0.00	0.00	0.00	0.00
CRC (4,130)	1.02	0.19	0.34	0.07	0.02	0.00	0.07	0.02	0.12	0.00	0.00	0.10	0.00	0.02	0.02	0.05	0.02	0.00	0.02	0.00	0.00	0.00	0.00	0.00	0.00
LUC (16,040)	7.11	4.20	1.28	1.16	0.17	0.01	0.02	0.11	0.07	0.04	0.02	0.01	0.01	0.02	0.01	0.01	0.00	0.00	0.01	0.02	0.00	0.01	0.01	0.01	0.00
All cancer (28,077)	4.72	2.50	0.84	0.71	0.18	0.14	0.04	0.11	0.13	0.03	0.04	0.03	0.01	0.01	0.01	0.01	0.01	0.00	0.01	0.01	0.01	0.01	0.01	0.01	0.00

RTK, receptor tyrosine kinase; LUC, lung cancer; CRC, colorectal cancer; GAC, gastric cancer; BRC, breast cancer; HEPC, hepatobiliary cancer; PAC, pancreatic cancer; OVC, ovarian cancer; STS, soft tissue sarcoma; CEC, cervical cancer; ESC, esophageal cancer; URC, urinary cancer; HNC, head and neck cancer; SKCM, skin cutaneous melanoma; NET, neuroendocrine tumors; PRC, prostate cancer; THC, thyroid cancer

Supplementary Table 2. Frequencies of RTK fusions across different cancer types in tumor specimens

	All RTK	ALK	RET	ROS1	FGFR3	FGFR2	FGFR1	EGFR	ERBB2	ERBB3	MET	NTRK1	NTRK3	PDGFRB	PDGFRA	FLT1	FLT3	FLT4	IGF1R	KDR	EPHA2	EPHA3	AXL	KIT	TEK
THC	0.67	0.00	100.00	0.00	0.00	0.00	0.00	0.00	0.00	0.00	0.00	0.00	0.00	0.00	0.00	0.00	0.00	0.00	0.00	0.00	0.00	0.00	0.00	0.00	0.00
PRC	0.08	0.00	0.00	0.00	0.00	0.00	100.00	0.00	0.00	0.00	0.00	0.00	0.00	0.00	0.00	0.00	0.00	0.00	0.00	0.00	0.00	0.00	0.00	0.00	0.00
NET	0.33	50.00	0.00	50.00	0.00	0.00	0.00	0.00	0.00	0.00	0.00	0.00	0.00	0.00	0.00	0.00	0.00	0.00	0.00	0.00	0.00	0.00	0.00	0.00	0.00
HNC	0.17	100.00	0.00	0.00	0.00	0.00	0.00	0.00	0.00	0.00	0.00	0.00	0.00	0.00	0.00	0.00	0.00	0.00	0.00	0.00	0.00	0.00	0.00	0.00	0.00
SKCM	0.33	0.00	0.00	25.00	0.00	25.00	0.00	0.00	0.00	0.00	0.00	0.00	0.00	0.00	0.00	0.00	0.00	25.00	0.00	0.00	25.00	0.00	0.00	0.00	0.00
ESC	0.17	0.00	50.00	0.00	50.00	0.00	0.00	0.00	0.00	0.00	0.00	0.00	0.00	0.00	0.00	0.00	0.00	0.00	0.00	0.00	0.00	0.00	0.00	0.00	0.00
CEC	0.58	14.29	0.00	0.00	28.57	0.00	0.00	0.00	57.14	0.00	0.00	0.00	0.00	0.00	0.00	0.00	0.00	0.00	0.00	0.00	0.00	0.00	0.00	0.00	0.00
URC	1.17	0.00	7.14	14.29	50.00	14.29	0.00	0.00	0.00	0.00	0.00	0.00	7.14	0.00	7.14	0.00	0.00	0.00	0.00	0.00	0.00	0.00	0.00	0.00	0.00
STS	0.58	71.43	14.29	14.29	0.00	0.00	0.00	0.00	0.00	0.00	0.00	0.00	0.00	0.00	0.00	0.00	0.00	0.00	0.00	0.00	0.00	0.00	0.00	0.00	0.00
OVC	0.67	37.50	12.50	12.50	0.00	0.00	0.00	12.50	12.50	0.00	12.50	0.00	0.00	0.00	0.00	0.00	0.00	0.00	0.00	0.00	0.00	0.00	0.00	0.00	0.00
PAC	0.17	0.00	50.00	0.00	0.00	0.00	0.00	0.00	50.00	0.00	0.00	0.00	0.00	0.00	0.00	0.00	0.00	0.00	0.00	0.00	0.00	0.00	0.00	0.00	0.00
HEPC	1.08	7.69	0.00	0.00	23.08	38.46	0.00	0.00	7.69	0.00	23.08	0.00	0.00	0.00	0.00	0.00	0.00	0.00	0.00	0.00	0.00	0.00	0.00	0.00	0.00
BRC	1.50	11.11	5.56	0.00	0.00	27.78	5.56	11.11	11.11	0.00	5.56	0.00	0.00	0.00	0.00	0.00	5.56	0.00	11.11	0.00	0.00	5.56	0.00	0.00	0.00
GAC	2.08	8.00	0.00	0.00	4.00	20.00	0.00	16.00	24.00	0.00	8.00	0.00	0.00	0.00	0.00	4.00	4.00	0.00	0.00	0.00	12.00	0.00	0.00	0.00	0.00
CRC	2.58	16.13	32.26	6.45	3.23	0.00	6.45	3.23	6.45	0.00	0.00	12.90	0.00	3.23	3.23	0.00	3.23	0.00	3.23	0.00	0.00	0.00	0.00	0.00	0.00
LUC	87.84	62.75	16.11	15.83	1.52	0.09	0.19	1.04	0.47	0.57	0.19	0.19	0.09	0.19	0.09	0.00	0.00	0.00	0.00	0.19	0.00	0.19	0.19	0.09	0.00
All tissue	100.00	57.04	16.15	14.65	2.58	1.58	0.50	1.58	1.83	0.50	0.75	0.50	0.17	0.25	0.25	0.08	0.25	0.08	0.25	0.17	0.33	0.25	0.17	0.08	0.00

RTK, receptor tyrosine kinase; LUC, lung cancer; CRC, colorectal cancer; GAC, gastric cancer; BRC, breast cancer; HEPC, hepatobiliary cancer; PAC, pancreatic cancer; OVC, ovarian cancer; STS, soft tissue sarcoma; CEC, cervical cancer; ESC, esophageal cancer; URC, urinary cancer; HNC, head and neck cancer; SKCM, skin cutaneous melanoma; NET, neuroendocrine tumors; PRC, prostate cancer; THC, thyroid cancer

Supplementary Table 3. Frequencies of RTK across different cancer types in cfDNA samples.

	All RTK	ALK	RET	ROS1	FGFR3	FGFR2	FGFR1	EGFR	ERBB2	ERBB3	MET	NTRK1	NTRK3	PDGFRB	PDGFRA	FLT1	FLT3	FLT4	IGF1R	KDR	EPHA2	EPHA3	AXL	KIT	TEK
THC	0.00	0.00	0.00	0.00	0.00	0.00	0.00	0.00	0.00	0.00	0.00	0.00	0.00	0.00	0.00	0.00	0.00	0.00	0.00	0.00	0.00	0.00	0.00	0.00	0.00
PRC	0.21	100.00	0.00	0.00	0.00	0.00	0.00	0.00	0.00	0.00	0.00	0.00	0.00	0.00	0.00	0.00	0.00	0.00	0.00	0.00	0.00	0.00	0.00	0.00	0.00
NET	0.85	50.00	50.00	0.00	0.00	0.00	0.00	0.00	0.00	0.00	0.00	0.00	0.00	0.00	0.00	0.00	0.00	0.00	0.00	0.00	0.00	0.00	0.00	0.00	0.00
HNC	0.21	0.00	0.00	0.00	100.00	0.00	0.00	0.00	0.00	0.00	0.00	0.00	0.00	0.00	0.00	0.00	0.00	0.00	0.00	0.00	0.00	0.00	0.00	0.00	0.00
SKCM	0.00	0.00	0.00	0.00	0.00	0.00	0.00	0.00	0.00	0.00	0.00	0.00	0.00	0.00	0.00	0.00	0.00	0.00	0.00	0.00	0.00	0.00	0.00	0.00	0.00
ESC	0.43	0.00	0.00	0.00	50.00	0.00	0.00	50.00	0.00	0.00	0.00	0.00	0.00	0.00	0.00	0.00	0.00	0.00	0.00	0.00	0.00	0.00	0.00	0.00	0.00
CEC	0.43	0.00	50.00	50.00	0.00	0.00	0.00	0.00	0.00	0.00	0.00	0.00	0.00	0.00	0.00	0.00	0.00	0.00	0.00	0.00	0.00	0.00	0.00	0.00	0.00
URC	0.64	0.00	0.00	0.00	66.70	33.30	0.00	0.00	0.00	0.00	0.00	0.00	0.00	0.00	0.00	0.00	0.00	0.00	0.00	0.00	0.00	0.00	0.00	0.00	0.00
STS	0.21	0.00	0.00	100.00	0.00	0.00	0.00	0.00	0.00	0.00	0.00	0.00	0.00	0.00	0.00	0.00	0.00	0.00	0.00	0.00	0.00	0.00	0.00	0.00	0.00
OVC	0.00	0.00	0.00	0.00	0.00	0.00	0.00	0.00	0.00	0.00	0.00	0.00	0.00	0.00	0.00	0.00	0.00	0.00	0.00	0.00	0.00	0.00	0.00	0.00	0.00
PAC	0.00	0.00	0.00	0.00	0.00	0.00	0.00	0.00	0.00	0.00	0.00	0.00	0.00	0.00	0.00	0.00	0.00	0.00	0.00	0.00	0.00	0.00	0.00	0.00	0.00
HEPC	0.43	0.00	0.00	0.00	0.00	0.00	0.00	100.00	0.00	0.00	0.00	0.00	0.00	0.00	0.00	0.00	0.00	0.00	0.00	0.00	0.00	0.00	0.00	0.00	0.00
BRC	2.13	22.22	0.00	11.11	0.00	0.00	11.11	11.11	22.22	0.00	0.00	11.11	0.00	0.00	0.00	0.00	0.00	0.00	0.00	0.00	0.00	0.00	0.00	0.00	11.11
GAC	6.40	3.33	10.00	3.33	10.00	60.00	3.33	3.33	6.67	0.00	0.00	0.00	0.00	0.00	0.00	0.00	0.00	0.00	0.00	0.00	0.00	0.00	0.00	0.00	0.00
CRC	3.84	27.78	33.33	5.56	0.00	0.00	5.56	0.00	16.67	0.00	0.00	0.00	0.00	0.00	0.00	11.11	0.00	0.00	0.00	0.00	0.00	0.00	0.00	0.00	0.00
LUC	84.22	55.44	23.29	12.41	3.04	0.25	0.25	1.77	1.77	0.25	0.25	0.00	0.00	0.25	0.00	0.25	0.00	0.00	0.25	0.25	0.00	0.00	0.00	0.25	0.00
All ctDNA	100.00	49.04	22.17	11.51	4.05	4.26	0.85	2.56	2.99	0.21	0.21	0.43	0.00	0.21	0.00	0.64	0.00	0.00	0.21	0.21	0.00	0.00	0.00	0.21	0.21

RTK, receptor tyrosine kinase; LUC, lung cancer; CRC, colorectal cancer; GAC, gastric cancer; BRC, breast cancer; HEPC, hepatobiliary cancer; PAC, pancreatic cancer; OVC, ovarian cancer; STS, soft tissue sarcoma; CEC, cervical cancer; ESC, esophageal cancer; URC, urinary cancer; HNC, head and neck cancer; SKCM, skin cutaneous melanoma; NET, neuroendocrine tumors; PRC, prostate cancer; THC, thyroid cancer

Supplementary Table 4. Concomitant driver mutations in RTK fusion carriers

Patient ID	Cancer type	Fusion RTK	Fusion gene	Clonality of fusion gene	concomitant driver gene	Driver mutation	Clonality of concomitant drivers
PT242	Lung	ALK	EML4-ALK V1	clonal	KRAS	Q61H	subclonal
PT852	Lung	ALK	EML4-ALK V3	clonal	BRCA2	G267fs	subclonal
PT1293	Lung	ALK	ARMC9-ALK	clonal	BRCA2	Q3047*	clonal
PT504	Lung	ALK	EML4-ALK V1	clonal	PTEN	E288fs	clonal
PT390	Lung	RET	KIF5B-RET	subclonal	NRAS	G12D	subclonal
PT787	Lung	RET	CCDC6-RET	subclonal	EGFR	ET710delinsD (exon 18)	subclonal
PT1309	Lung	RET	VSTM4-RET	clonal	EGFR	L858R	clonal
					PIK3CA	H1047R	subclonal
PT750	Lung	RET	DCLRE1C-RET	subclonal	EGFR	N771delinsNG (exon 20)	subclonal
PT246	Lung	RET	KIF5B-RET	clonal	BRCA1	E670fs	subclonal
PT16	Lung	RET	KIF5B-RET	clonal	PTEN	V317fs	subclonal
PT500	Lung	RET	CCDC6-RET	subclonal	PTEN	D22fs	subclonal
PT576	CRC	ROS1	GOPC-ROS1	subclonal	BRCA2	T3030fs	subclonal
PT790	Lung	ROS1	SDC4-ROS1	subclonal	PIK3CA	H1047R	subclonal
PT1266	Hepatobiliary	FGFR2	FGFR2-PROB1	clonal	NRAS	G12V	subclonal
					KRAS	G12V	clonal
PT896	Breast	FGFR2	FGFR2-SHTN1	clonal	PIK3CA	H1047R	clonal
PT535	Lung	FGFR3	FGFR3-TACC3	clonal	KRAS	G13D	subclonal
PT783	Gastric	FGFR3	FGFR3-TACC3	subclonal	KRAS	A146V	subclonal
					KRAS	Q61H	subclonal
					KRAS	Q61R	subclonal
PT518	Ovarian	MET	SLC25A19-MET	clonal	KRAS	G12V	clonal
