# Supplementary material for: Landscape of potentially targetable receptor tyrosine kinase fusions in diverse cancers by DNA-based profiling
Source: NPJ Precis Oncol. 2022 Nov 11;6:84. doi: 10.1038/s41698-022-00325-0 (PMC9652465; doi:10.1038/s41698-022-00325-0)
Supplement: Supplementary file 2 — REPORTING SUMMARY [file 41698_2022_325_MOESM2_ESM.pdf]

nature portfolio

Corresponding author(s): Zhe Wang and Zhanhong Chen

Last updated by author(s): Jul 3, 2022

Reporting Summary

Nature Portfolio wishes to improve the reproducibility of the work that we publish. This form provides structure for consistency and transparency in reporting. For further information on Nature Portfolio policies, see our Editorial Policies and the Editorial Policy Checklist.

Statistics

For all statistical analyses, confirm that the following items are present in the figure legend, table legend, main text, or Methods section.

n/a	Confirmed	
☐	☒	The exact sample size (*n*) for each experimental group/condition, given as a discrete number and unit of measurement
☐	☒	A statement on whether measurements were taken from distinct samples or whether the same sample was measured repeatedly
☐	☒	The statistical test(s) used AND whether they are one- or two-sided<br>*Only common tests should be described solely by name; describe more complex techniques in the Methods section.*
☐	☒	A description of all covariates tested
☐	☒	A description of any assumptions or corrections, such as tests of normality and adjustment for multiple comparisons
☐	☒	A full description of the statistical parameters including central tendency (e.g. means) or other basic estimates (e.g. regression coefficient) AND variation (e.g. standard deviation) or associated estimates of uncertainty (e.g. confidence intervals)
☐	☒	For null hypothesis testing, the test statistic (e.g. *F*, *t*, *r*) with confidence intervals, effect sizes, degrees of freedom and *P* value noted<br>*Give P values as exact values whenever suitable.*
☐	☒	For Bayesian analysis, information on the choice of priors and Markov chain Monte Carlo settings
☐	☒	For hierarchical and complex designs, identification of the appropriate level for tests and full reporting of outcomes
☐	☒	Estimates of effect sizes (e.g. Cohen's *d*, Pearson's *r*), indicating how they were calculated

Our web collection on statistics for biologists contains articles on many of the points above.

Software and code

Policy information about availability of computer code

Data collection	no software was used.
Data analysis	All statistical analyses were done in R (v.3.5.2) using commercially available codes

For manuscripts utilizing custom algorithms or software that are central to the research but not yet described in published literature, software must be made available to editors and reviewers. We strongly encourage code deposition in a community repository (e.g. GitHub). See the Nature Portfolio guidelines for submitting code & software for further information.

Data

Policy information about availability of data

All manuscripts must include a data availability statement. This statement should provide the following information, where applicable:

- Accession codes, unique identifiers, or web links for publicly available datasets
- A description of any restrictions on data availability
- For clinical datasets or third party data, please ensure that the statement adheres to our policy

The datasets generated during and/or analysed during the current study are available from the corresponding author on reasonable request.

Human research participants

Policy information about studies involving human research participants and Sex and Gender in Research.

Reporting on sex and gender	Sex were considered in the study design based on self-reporting. male sex=627, female sex=678
Population characteristics	Patient age, genetics and treatment histories were considered in the study. Mutations or clinical outcomes were analyzed in association with age and treatment histories.
Recruitment	Patients routinely treated at multiple hospitals in different regions from 2015-2020 were retrospectively analyzed. No potential selection bias should exist.
Ethics oversight	Informed written consent was obtained from each subject or the subject's family member upon sample collection according to the protocols approved by the ethics committee of each hospital.

Note that full information on the approval of the study protocol must also be provided in the manuscript.

Field-specific reporting

Please select the one below that is the best fit for your research. If you are not sure, read the appropriate sections before making your selection.

☒ Life sciences ☐ Behavioural & social sciences ☐ Ecological, evolutionary & environmental sciences

For a reference copy of the document with all sections, see nature.com/documents/nr-reporting-summary-flat.pdf

Life sciences study design

All studies must disclose on these points even when the disclosure is negative.

Sample size	A total of 28,077 patients routinely treated at multiple hospitals in different regions from 2015-2020 were retrospectively analyzed for RTK fusion positivity. A total of 1,324 RTK fusion-positive patients were identified. Such a large cohort should be sufficient.
Data exclusions	No data were excluded from the analyses.
Replication	Measures taken to ensure the reproducibility of the findings include: large sample size, FDR-multiple testing corrections, co-variate analyses.
Randomization	No randomization was performed for the retrospective analysis. Covariates, including age and sex and cancer types, were analyzed in relation to mutations and/or clinical outcomes.
Blinding	Blinding was not relevant due to the retrospective nature of the study.

Reporting for specific materials, systems and methods

We require information from authors about some types of materials, experimental systems and methods used in many studies. Here, indicate whether each material, system or method listed is relevant to your study. If you are not sure if a list item applies to your research, read the appropriate section before selecting a response.

Materials & experimental systems

n/a	Involved in the study	
☒	☐	Antibodies
☒	☐	Eukaryotic cell lines
☒	☐	Palaeontology and archaeology
☒	☐	Animals and other organisms
☒	☐	Clinical data
☒	☐	Dual use research of concern

Methods

n/a	Involved in the study	
☒	☐	ChIP-seq
☒	☐	Flow cytometry
☒	☐	MRI-based neuroimaging
